# Supplementary material for: Analysis of Movement Variability in Cycling: An Exploratory Study
Source: Sensors (Basel). 2023 May 22;23(10):4972. doi: 10.3390/s23104972 (PMC10223996; doi:10.3390/s23104972)
Supplement: Supplementary file 1 [file sensors-23-04972-s001.zip › sensors-2371126-supplementary.pdf]

Table S1. IMU head, thorax acceleration state space reconstruction

		EDim		ELag	
**Segment/Joint (Axis)**	**Interval**	**Min**	**Max**	**Min**	**Max**
Head (X)	1	4	4	10	10
	2	4	4	10	10
	3	4	4	10	10
	4	4	4	10	10
	5	4	4	10	10
Head (Y)	1	4	4	10	10
	2	4	4	10	10
	3	4	4	10	10
	4	4	4	10	10
	5	4	4	10	10
Head (Z)	1	4	4	10	10
	2	4	4	10	10
	3	4	4	10	10
	4	4	4	5	10
	5	3	4	10	10
Thorax (X)	1	4	4	10	10
	2	4	4	10	10
	3	4	4	10	10
	4	4	4	10	10
	5	4	4	10	10
Thorax (Y)	1	4	4	10	10
	2	4	4	10	10
	3	4	4	10	10
	4	4	4	10	10
	5	4	4	10	10
Thorax (Z)	1	4	4	9	10
	2	4	4	9	10
	3	4	4	8	10
	4	4	4	9	10
	5	4	4	8	10

Edim = embedding dimension, Elag = time delay, Min = minimum, Max = maximum

Table S2. IMU pelvis acceleration state space reconstruction

		EDim		Elag	
**Segment/Joint (Axis)**	**Interval**	**Min**	**Max**	**Min**	**Max**
Pelvis (X)	1	4	4	10	10
	2	4	4	10	10
	3	4	4	10	10
	4	4	4	10	10
	5	4	4	10	10
Pelvis (Y)	1	4	4	10	10
	2	4	4	10	10
	3	4	4	10	10
	4	4	4	10	10
	5	4	4	10	10
Pelvis (Z)	1	4	4	10	10
	2	4	4	10	10
	3	4	4	10	10
	4	4	4	10	10
	5	4	4	10	10

Edim = embedding dimension, Elag = time delay, Min = minimum, Max = maximum

Table S3. IMU shank acceleration state space reconstruction

		EDim		ELag	
**Segment/Joint (Axis)**	**Interval**	**Min**	**Max**	**Min**	**Max**
Left Shank (X)	1	4	4	10	10
	2	4	4	10	10
	3	4	4	10	10
	4	4	4	10	10
	5	4	4	10	10
Left Shank (Y)	1	4	4	10	10
	2	4	4	10	10
	3	4	4	10	10
	4	4	4	10	10
	5	4	4	10	10
Left Shank (Z)	1	4	4	10	10
	2	4	4	10	10
	3	4	4	9	10
	4	4	4	9	10
	5	4	4	10	10
Right Shank (X)	1	4	4	10	10
	2	4	4	10	10
	3	4	4	10	10
	4	4	4	10	10
	5	4	4	10	10
Right Shank (Y)	1	4	4	10	10
	2	4	4	10	10
	3	4	4	10	10
	4	4	4	10	10
	5	4	4	10	10
Right Shank (Z)	1	4	4	10	10
	2	4	4	9	10
	3	4	4	10	10
	4	4	4	10	10
	5	4	4	10	10

Edim = embedding dimension, Elag = time delay, Min = minimum, Max = maximum

Table S4. VICON Neck, thorax kinematic state space reconstruction

		EDim		ELag	
**Segment/Joint (Axis)**	**Interval**	**Min**	**Max**	**Min**	**Max**
Neck (X)	1	3	4	10	10
	2	3	4	10	10
	3	3	4	2	10
	4	3	4	9	10
	5	3	4	10	10
Neck (Y)	1	3	4	10	10
	2	3	4	10	10
	3	3	4	10	10
	4	3	4	10	10
	5	3	4	10	10
Neck (Z)	1	3	4	10	10
	2	3	4	10	10
	3	4	4	10	10
	4	3	4	10	10
	5	3	4	10	10
Thorax (X)	1	3	4	8	10
	2	3	4	8	10
	3	3	4	7	10
	4	4	4	8	10
	5	3	4	8	10
Thorax (Y)	1	3	4	10	10
	2	3	4	10	10
	3	3	4	10	10
	4	3	4	10	10
	5	3	4	10	10
Thorax (Z)	1	3	4	9	10
	2	3	4	10	10
	3	3	4	10	10
	4	3	4	10	10
	5	3	4	10	10

Edim = embedding dimension, Elag = time delay, Min = minimum, Max = maximum

Table S5. VICON pelvis kinematic state space reconstruction

		EDim		ELag	
Segment/Joint (Axis)	Interval	Min	Max	Min	Max
Pelvis (X)	1	3	4	10	10
	2	3	4	10	10
	3	3	4	9	10
	4	3	4	10	10
	5	3	4	10	10
Pelvis (Y)	1	3	4	7	10
	2	3	4	8	10
	3	3	4	9	10
	4	3	4	9	10
	5	3	4	9	10
Pelvis (Z)	1	3	4	10	10
	2	3	4	9	10
	3	3	4	9	10
	4	3	4	10	10
	5	3	4	10	10

Edim = embedding dimension, Elag = time delay, Min = minimum, Max = maximum

Table S6. VICON hip kinematics state space reconstruction

		EDim		ELag	
Segment/Joint (Axis)	Interval	Min	Max	Min	Max
Left Hip (X)	1	3	4	7	10
	2	3	4	7	10
	3	3	4	7	10
	4	4	4	7	10
	5	3	4	7	10
Left Hip (Y)	1	3	4	8	10
	2	3	4	7	10
	3	3	4	7	10
	4	3	4	8	10
	5	3	4	7	10
Left Hip (Z)	1	3	4	4	10
	2	3	4	4	10
	3	4	4	4	10
	4	3	4	4	10
	5	3	4	4	10
Right Hip (X)	1	4	4	7	10
	2	4	4	7	10
	3	4	4	6	10
	4	4	4	6	10
	5	4	4	6	10
Right Hip (Y)	1	3	4	8	10
	2	3	4	7	10
	3	3	4	7	10
	4	3	4	6	10
	5	3	4	7	10
Right Hip (Z)	1	3	3	4	10
	2	3	3	4	10
	3	3	3	4	10
	4	3	3	4	10
	5	3	3	4	10

Edim = embedding dimension, Elag = time delay, Min = minimum, Max = maximum

Table S7. VICON knee kinematics state space reconstruction

		EDim		ELag	
Segment/Joint (Axis)	Interval	Min	Max	Min	Max
Left Knee (X)	1	4	4	5	10
	2	4	4	5	10
	3	4	4	5	10
	4	4	4	5	10
	5	4	4	5	10
Left Knee (Y)	1	4	4	5	10
	2	4	4	5	10
	3	4	4	5	10
	4	4	4	5	10
	5	4	4	5	10
Left Knee (Z)	1	3	3	3	10
	2	3	3	3	10
	3	3	3	3	8
	4	3	3	3	10
	5	3	3	3	10
Right Knee (X)	1	4	4	6	10
	2	4	4	6	10
	3	4	4	6	10
	4	4	4	5	10
	5	4	4	5	10
Right Knee (Y)	1	3	4	6	10
	2	3	4	6	10
	3	3	4	5	10
	4	3	4	6	10
	5	3	4	5	10
Right Knee (Z)	1	3	3	3	10
	2	3	3	3	6
	3	3	3	3	5
	4	3	3	3	5
	5	3	3	3	8

Edim = embedding dimension, Elag = time delay, Min = minimum, Max = maximum

Table S8. VICON ankle kinematics state space reconstruction

		EDim		ELag	
**Segment/Joint (Axis)**	**Interval**	**Min**	**Max**	**Min**	**Max**
Left Ankle (X)	1	4	4	8	10
	2	4	4	8	10
	3	4	4	8	10
	4	4	4	7	10
	5	4	4	8	10
Left Ankle (Y)	1	4	4	5	10
	2	4	4	5	10
	3	4	4	5	10
	4	4	4	5	10
	5	4	4	5	10
Left Ankle (Z)	1	3	4	10	10
	2	3	4	10	10
	3	3	4	10	10
	4	3	4	10	10
	5	3	4	10	10
Right Ankle (X)	1	4	4	9	10
	2	3	4	9	10
	3	3	4	7	10
	4	4	4	7	10
	5	4	4	7	10
Right Ankle (Y)	1	4	4	7	10
	2	4	4	6	10
	3	4	4	7	10
	4	4	4	6	10
	5	4	4	6	10
Right Ankle (Z)	1	3	4	8	10
	2	3	4	8	10
	3	3	4	9	10
	4	3	4	9	10
	5	3	4	8	10

Edim = embedding dimension, Elag = time delay, Min = minimum, Max = maximum

Table S9: Reliability cycling performance variables

Value	Interval	Mean (SD)			$ICC_{(3,1)}$ (95%CI)			$ICC_{(3,1)}$ mean (95%CI)	TE (stzd) (LL/UL)			TE (stzd) mean (LL/UL)
		**Sn. 1**	**Sn. 2**	**Sn. 3**	**2-1**	**3-1**	**3-2**		**2-1**	**3-1**	**3-2**	
**P**	1	227.1 (72.4)	240.2 (74.1)	244.4 (68.1)	0.89 (0.67 – 0.97)	0.88 (0.64 – 0.96)	0.87 (0.61 – 0.96)	0.88 (0.73 – 0.96)	0.78 (0.56 – 1.33)	0.82 (0.58 – 1.39)	0.86 (0.61 – 1.47)	0.76 (0.61 – 1.07)
	2	199.0 (58.9)[†]	211.4 (66.1)[†]	213.8 (58.4)[†]	0.95 (0.82 – 0.98)	0.96 (0.88 – 0.99)	0.96 (0.88 – 0.99)	0.96 (0.90 – 0.99)	0.54 (0.38 – 0.92)	0.43 (0.31 – 0.73)	0.44 (0.31 – 0.74)	0.46 (0.37 – 0.65)
	3	193.4 (54.2)[†]	206.3 (62.3)[†]	206.3 (60.0)[†]	0.97 (0.89 – 0.99)	0.94 (0.81 – 0.98)	0.93 (0.79 – 0.98)	0.95 (0.87 – 0.98)	0.41 (0.29 – 0.70)	0.56 (0.40 – 0.95)	0.60 (0.42 – 1.01)	0.51 (0.41 – 0.72)
	4	196.7 (52.2)[†]	204.7 (60.7)[†]	205.0 (61.0)[†]	0.97 (0.91 – 0.99)	0.94 (0.82 – 0.98)	0.95 (0.84 – 0.99)	0.96 (0.89 – 0.99)	0.38 (0.27 – 0.65)	0.55 (0.39 – 0.94)	0.51 (0.36 – 0.87)	0.47 (0.38 – 0.66)
	5	207.9 (46.0)[†]	210.7 (55.7)[†]	220.2 (60.3)[†]	0.95 (0.83 - 0.98)	0.94 (0.81 - 0.98)	0.95 (0.84-0.99)	0.95 (0.87 – 0.98)	0.52 (0.37 – 0.89)	0.57 (0.40 – 0.96)	0.51 (0.36 – 0.87)	0.52 (0.41 – 0.72)
**C**	1	90.0 (10.0)	91.2 (9.8)	92.4 (8.0)	0.79 (0.43 – 0.94)	0.78 (0.40 – 0.93)	0.82 (0.48 – 0.94)	0.80 (0.57 – 0.93)	1.13 (0.80 – 1.93)	1.17 (0.83 – 1.98)	1.06 (0.75 – 1.79)	0.98 (0.78 – 1.37)
	2	86.3 (8.3)[†]	87.3 (8.9)[†]	88.4 (5.6)[†]	0.85 (0.56 – 0.95)	0.86 (0.58 – 0.96)	0.86 (0.59 – 0.96)	0.86 (0.68 – 0.95)	0.93 (0.66 – 1.58)	0.90 (0.64 – 1.54)	0.89 (0.63 – 1.52)	0.83 (0.66 – 1.16)
	3	85.4 (7.9)[†]	86.7 (8.3)[†]	87.1 (5.1)[†]	0.89 (0.66 – 0.97)	0.80 (0.44 – 0.94)	0.75 (0.33 – 0.92)	0.82 (0.62 – 0.94)	0.79 (0.56 – 1.34)	1.11 (0.79 – 1.89)	1.29 (0.91 – 2.18)	0.92 (0.73 – 1.29)
	4	86.1 (7.6)[†]	86.5 (7.8)[†]	87.0 (5.3)[†]	0.92 (0.74 – 0.98)	0.83 (0.51 – 0.95)	0.83 (0.51 – 0.95)	0.87 (0.70 – 0.95)	0.67 (0.47 – 1.14)	1.01 (0.72 – 1.72)	1.01 (0.71 – 1.71)	0.80 (0.64 – 1.13)
	5	88.0 (5.8)	87.5 (6.7)[†]	89.3 (5.6)[†]	0.93 (0.77 – 0.98)	0.86 (0.60 – 0.96)	0.89 (0.66 – 0.97)	0.90 (0.76 – 0.97)	0.63 (0.44 – 1.06)	0.88 (0.63 – 1.50)	0.80 (0.57 – 1.35)	0.71 (0.57 – 1.00)
**F**	1	139.3 (32.4)[†]	145.2 (33.3)[†]	146.6 (30.8)[†]	0.90 (0.70 – 0.97)	0.91 (0.72 – 0.97)	0.91 (0.72 – 0.97)	0.91 (0.79 – 0.97)	0.73 (0.52 – 1.24)	0.70 (0.50 – 1.19)	0.70 (0.50 – 1.19)	0.67 (0.53 – 0.94)
	2	128.1 (27.9)[†]	133.7 (30.8)[†]	134.7 (28.6)[†]	0.95 (0.83 – 0.98)	0.97 (0.90 – 0.99)	0.97 (0.91 – 0.99)	0.96 (0.91 – 0.99)	0.52 (0.37 – 0.89)	0.39 (0.28 – 0.66)	0.38 (0.27 – 0.65)	0.43 (0.34 – 0.60)
	3	125.9 (26.2)[†]	131.8 (29.3)[†]	131.8 (29.4)[†]	0.97 (0.89 – 0.99)	0.96 (0.86 – 0.99)	0.95 (0.84 – 0.99)	0.96 (0.90 – 0.99)	0.41 (0.29 – 0.69)	0.47 (0.34 – 0.81)	0.52 (0.37 – 0.88)	0.46 (0.36 – 0.64)
	4	127.3 (25.3)[†]	131.2 (28.7)[†]	131.1 (29.9)[†]	0.96 (0.88 – 0.99)	0.96 (0.86 – 0.99)	0.96 (0.87 – 0.99)	0.96 (0.90 – 0.99)	0.43 (0.31 – 0.73)	0.48 (0.34 – 0.82)	0.45 (0.32 – 0.77)	0.44 (0.35 – 0.62)
	5	132.0 (23.7)[†]	133.9 (26.6)[†]	137.1 (29.4)[†]	0.94 (0.81 – 0.98)	0.95 (0.85 – 0.99)	0.96 (0.87 – 0.99)	0.95 (0.89 – 0.98)	0.56 (0.40 – 0.96)	0.49 (0.35 – 0.83)	0.45 (0.32 – 0.77)	0.49 (0.39 – 0.68)
**P/m**	1	3.0 (1.0)	3.1 (0.9)	3.2 (0.9)	0.87 (0.61 – 0.96)	0.90 (0.69 – 0.97)	0.86 (0.58 – 0.96)	0.88 (0.72 – 0.96)	0.87 (0.62 – 1.48)	0.75 (0.53 – 1.28)	0.90 (0.64 – 1.54)	0.78 (0.62 – 1.09)
	2	2.6 (0.7)[†]	2.7 (0.8)[†]	2.8 (0.7)[†]	0.93 (0.78 – 0.98)	0.96 (0.87 – 0.99)	0.95 (0.84 – 0.99)	0.95 (0.87 – 0.98)	0.61 (0.43 – 1.03)	0.45 (0.32 – 0.77)	0.51 (0.36 – 0.87)	0.51 (0.41 – 0.72)
	3	2.5 (0.6)[†]	2.7 (0.7)[†]	2.7 (0.6)[†]	0.96 (0.86 – 0.99)	0.92 (0.74 – 0.98)	0.89 (0.67 – 0.97)	0.92 (0.82 – 0.97)	0.48 (0.34 – 0.82)	0.67 (0.47 – 1.13)	0.78 (0.55 – 1.32)	0.62 (0.49 – 0.87)

Value	Interval	Mean (SD)			ICC$_{(3,1)}$ (95%CI)			ICC$_{(3,1)}$ mean (95%CI)	TE (stzd) (LL/UL)			TE (stzd) mean (LL/UL)
		Sn. 1	Sn. 2	Sn. 3	2-1	3-1	3-2		2-1	3-1	3-2	
	4	2.6 (0.6)†	2.7 (0.7)†	2.6 (0.6)†	0.96 (0.87 – 0.99)	0.92 (0.75 – 0.98)	0.92 (0.74 – 0.98)	0.93 (0.84 – 0.98)	0.46 (0.33 – 0.79)	0.66 (0.46 – 1.11)	0.67 (0.47 – 1.13)	0.57 (0.46 – 0.81)
	5	2.7 (0.5)†	2.7 (0.6)†	2.8 (0.6)†	0.92 (0.75 – 0.98)	0.91 (0.72 – 0.97)	0.92 (0.75 – 0.98)	0.92 (0.81 – 0.97)	0.66 (0.47 – 1.13)	0.70 (0.50 – 1.19)	0.66 (0.47 – 1.12)	0.64 (0.51 – 0.89)

P = Power (watts), C = Cadence (revolutions/min), F = Force (Newtons), P/m = Power / mass (Watts/kg); Sn = session; SD = standard deviation; ICC = intraclass correlation coefficient; CI = confidence interval; TE (stzd) = standardized typical error; LL = lower limit; UL = upper limit.

† < 0.05 vs. Interval 1

Table S10. Reliability of the LyE for head acceleration

ACC	Interval	Mean (SD)			ICC(3,1) (95%CI)			ICC(3,1) mean (95%CI)	TE (stzd) (LL/UL)			TE (stzd) mean (LL/UL)
		Sn. 1	Sn. 2	Sn. 3	2-1	3-1	3-2		2-1	3-1	3-2	
Hx	1	1.4 (0.3)	1.4 (0.3)	1.3 (0.2)	0.72 (0.27-0.91)	0.74 (0.-32-0.92)	0.89 (0.67-0.97)	0.77 (0.53-0.92)	1.39 (0.98-2.36)	1.31 (0.93-2.22)	0.78 (0.55-1.32)	1.03 (0.82-1.44)
	2	1.4 (0.2)	1.5 (0.2)[a]	1.4 (0.2)	0.76 (0.35-0.92)	0.80 (0.45-0.94)	0.85 (0.55-0.95)	0.80 (0.58-0.93)	1.26 (0.89-2.14)	1.10 (0.78-1.87)	0.95 (0.67-1.61)	0.97 (0.77-1.35)
	3	1.5 (0.2)[a]	1.5 (0.2)[a]	1.5 (0.2)[a]	0.83 (0.52 – 0.95)	0.91 (0.72 – 0.97)	0.93 (0.77 – 0.98)	0.89 (0.75 – 0.96)	1.00 (0.71 – 1.69)	0.71 (0.50 – 1.21)	0.62 (0.44 – 1.06)	0.73 (0.58 – 1.02)
	4	1.6 (0.3)[ab]	1.6 (0.3)[ab]	1.5 (0.3)[ab]	0.86 (0.58 – 0.96)	0.90 (0.69 – 0.97)	0.96 (0.86 – 0.99)	0.91 (0.78 – 0.97)	0.91 (0.64 – 1.54)	0.75 (0.53 – 1.27)	0.48 (0.34 – 0.82)	0.68 (0.54 – 0.95)
	5	1.7 (0.2)[abc]	1.7 (0.2)[abc]	1.7 (0.2)[abc]	0.88 (0.65 – 0.96)	0.77 (0.39 – 0.93)	0.92 (0.75 – 0.98)	0.87 (0.70 – 0.95)	0.81 (0.58 – 1.38)	0.99 (0.70-1.68)	0.65 (0.46 – 1.11)	0.80 (0.64 – 1.13)
Hy	1	1.2 (0.2)	1.2 (0.3)	1.3 (0.2)	0.76 (0.35 – 0.92)	0.90 (0.69 – 0.97)	0.80 (0.45 – 0.94)	0.81 (0.60 – 0.93)	1.26 (0.89 – 2.13)	0.75 (0.53 – 1.28)	1.10 (0.78 – 1.87)	0.94 (0.75 – 1.32)
	2	1.3 (0.2)	1.3 (0.2)	1.3 (0.2)	0.69 (0.23 – 0.90)	0.79 (0.42 – 0.93)	0.77 (0.37 – 0.93)	0.75 (0.49 – 0.91)	1.46 (1.04 – 2.48)	1.15 (0.81 – 1.95)	1.22 (0.87 – 2.08)	1.08 (0.86 – 1.52)
	3	1.3 (0.2)	1.4 (0.2)	1.4 (0.2)[a]	0.72 (0.28 – 0.91)	0.76 (0.36 – 0.92)	0.90 (0.70 – 0.97)	0.80 (0.59 – 0.93)	1.37 (0.97 – 2.32)	1.24 (0.88 – 2.10)	0.74 (0.52 – 1.25)	0.96 (0.76 – 1.34)
	4	1.4 (0.2)[ab]	1.5 (0.3)[ab]	1.5 (0.3)[ab]	0.70 (0.25 – 0.90)	0.69 (0.23 – 0.90)	0.75 (0.33 – 0.92)	0.72 (0.45 – 0.90)	1.43 (1.01 – 2.42)	1.46 (1.46 – 2.27)	1.28 (0.91 – 2.18)	1.13 (0.90 – 1.59)
	5	1.5 (0.2)[abc]	1.6 (0.3)[abc]	1.6 (0.4)[abcd]	0.76 (0.35 – 0.92)	0.65 (0.15 – 0.88)	0.88 (0.64 – 0.96)	0.78 (0.55 – 0.92)	1.25 (0.88 – 2.12)	1.62 (1.15 – 2.75)	0.81 (0.58 – 1.38)	1.01 (0.81 – 1.42)
Hz	1	1.3 (0.3)	1.4 (0.4)	1.4 (0.5)	0.53 (-0.03 – 0.84)	0.48 (-0.10–0.81)	0.75 (0.33-0.92)	0.61 (0.30 – 0.85)	2.03 (1.44 – 3.45)	2.14 (1.52 – 3.64)	1.29 (0.91 – 2.19)	1.31 (1.04 – 1.84)
	2	1.4 (0.2)	1.5 (0.3)	1.5 (0.5)	0.68 (0.21 – 0.90)	0.56 (0.02 – 0.85)	0.68 (0.20 – 0.90)	0.64 (0.34 – 0.86)	1.50 (1.06 – 2.55)	1.91 (1.35-3.24)	1.52 (1.07 – 2.57)	1.27 (1.01 – 1.78)
	3	1.5 (0.3)[a]	1.5 (0.4)	1.6 (0.6)	0.68 (0.20 – 0.89)	0.65 (0.15 – 0.89)	0.88 (0.64 – 0.96)	0.76 (0.52 – 0.91)	1.52 (1.08 – 2.58)	1.60 (1.13-2.72)	0.82 (0.58 – 1.39)	1.05 (0.84 – 1.47)
	4	1.6 (0.3)[a]	1.6 (0.6)[a]	1.6 (0.6)	0.64 (0.13 – 0.88)	0.70 (0.25 – 0.91)	0.84 (0.54 – 0.95)	0.75 (0.50 – 0.91)	1.65 (1.17 – 2.81)	1.42 (1.01 – 2.42)	0.96 (0.68 – 1.63)	1.07 (0.86 – 1.50)
	5	1.7 (0.3)[ab]	1.7 (0.5)[a]	1.7 (0.6)[ab]	0.67 (0.18– 0.89)	0.63 (0.12 – 0.88)	0.93 (0.77 – 0.98)	0.78 (0.54 – 0.92)	1.55 (1.10 – 2.63)	1.66 (1.18 – 2.82)	0.63 (0.45 – 1.07)	1.02 (0.81 – 1.42)

ACC = acceleration; Hx = head longitudinal; Hy = head medio-lateral; Hz = head anterior-posterior; Sn = session; SD = standard deviation; ICC = intraclass correlation coefficient; CI = confidence interval; TE (stzd) = standardized typical error; LL = lower limit; UL = upper limit.

a < 0.05 vs interval 1; b < 0.05 vs. interval 2; c < 0.05 vs. interval 3; d < 0.05 vs. interval 4

Table S11. Reliability of the LyE for thorax acceleration

ACC	Interval	Mean (SD)			ICC$_{(3,1)}$ (95%CI)			ICC$_{(3,1)}$ mean (95%CI)	TE (stzd) (LL/UL)			TE (stzd) mean (LL/UL)
		Sn. 1	Sn. 2	Sn. 3	2-1	3-1	3-2		2-1	3-1	3-2	
Tx	1	1.2 (0.3)	1.2 (0.2)	1.2 (0.2)	0.73 (0.29 – 0.91)	0.80 (0.44 – 0.94)	0.91 (0.72 – 0.97)	0.81 (0.60 – 0.93)	1.36 (0.96 – 2.30)	1.12 (0.79 – 1.90)	0.70 (0.50 – 1.20)	0.95 (0.75 – 1.33)
	2	1.3 (0.3)	1.3 (0.2)	1.3 (0.3)	0.85 (0.57 – 0.96)	0.90 (0.68 – 0.97)	0.92 (0.75 – 0.98)	0.89 (0.75 – 0.96)	0.92 (0.65 – 1.57)	0.76 (0.54 – 1.29)	0.66 (0.47 – 1.12)	0.72 (0.58 – 1.01)
	3	1.3 (0.3$^{)}$	1.3 (0.2)	1.3 (0.3)	0.89 (0.67 – 0.97)	0.87 (0.61 – 0.96)	0.93 (0.76 – 0.98)	0.89 (0.76 – 0.96)	0.78 (0.55 – 1.33)	0.87 (0.61 – 1.47)	0.64 (0.45 – 1.08)	0.72 (0.57 – 1.01)
	4	1.4 (0.3)$_{a}$	1.3 (0.2)$_{ab}$	1.3 (0.3)^{ab}	0.86 (0.58 – 0.96)	0.86 (0.59 – 0.96)	0.89 (0.66 – 0.97)	0.87 (0.71 – 0.96)	0.90 (0.64 – 1.53)	0.89 (0.63 – 1.52)	0.79 (0.56 – 1.34)	0.79 (0.63 – 1.11)
	5	1.4 (0.3)$_{abc}$	1.4 (0.3)$_{abc}$	1.4 (0.3)$_{abc}$	0.93 (0.78 – 0.98)	0.85 (0.56 – 0.95)	0.91 (0.73 – 0.97)	0.90 (0.76 – 0.97)	0.61 (0.43 – 1.04)	0.94 (0.67 – 1.60)	0.69 (0.49 – 1.16)	0.71 (0.57 – 1.00)
Ty	1	1.4 (0.2)	1.4 (0.2)	1.3 (0.2)	0.50 (-0.07-0.82)	0.68 (0.20 – 0.89)	0.65 (0.16 – 0.89)	0.60 (0.29 – 0.85)	2.17 (1.54 – 3.69)	1.52 (1.08 – 2.58)	1.60 (1.13 – 2.72)	1.33 (1.05 – 1.86)
	2	1.4 (0.3)	1.4 (0.2)	1.4 (0.2)	0.70 (0.24 – 0.90)	0.80 (0.45 – 0.94)	0.89 (0.66 – 0.97)	0.79 (0.56 – 0.93)	1.44 (1.02 – 2.45)	1.09 (0.78 – 1.86)	0.80 (0.57 – 1.36)	1.00 (0.79 – 1.40)
	3	1.5 (0.3)	1.5 (0.2)	1.4 (0.2)	0.77 (0.37 – 0.93)	0.73 (0.30 – 0.91)	0.89 (0.67 – 0.97)	0.79 (0.56 – 0.92)	1.23 (0.87 – 2.08)	1.33 (0.94 – 2.26)	0.78 (0.55 – 1.32)	1.00 (0.80 – 1.40)
	4	1.5 (0.3)$_{abc}$	1.5 (0.2)$_{ab}$	1.5 (0.2)^{ab}	0.72 (0.27 – 0.91)	0.76 (0.35 – 0.92)	0.88 (0.64 – 0.96)	0.78 (0.54 – 0.92)	1.38 (0.98 – 2.34)	1.25 (0.88 – 2.12)	0.82 (0.58 – 1.40)	1.02 (0.81 – 1.42)
	5	1.6 (0.3)$_{abc}$	1.6 (0.3)$_{abc}$	1.6 (0.2)$_{abc}$	0.88 (0.63 – 0.96)	0.78 (0.41 – 0.93)	0.93 (0.79 – 0.98)	0.86 (0.69 – 0.95)	0.84 (0.59 – 1.42)	1.17 (0.83 – 1.98)	0.60 (0.43 – 1.02)	0.81 (0.65 – 1.14)
Tz	1	1.6 (0.6)	1.7 (0.5)	1.7 (0.5)	0.50 (-0.07-0.83)	0.58 (0.03 – 0.86)	0.92 (0.74 – 0.98)	0.65 (0.35 – 0.87)	2.16 (1.53 – 3.67)	1.87 (1.33 – 3.18)	0.67 (0.48 – 1.14)	1.26 (1.00 – 1.76)
	2	1.8 (0.4)$_{a}$	1.8 (0.4)	1.8 (0.5)	0.74 (0.32 – 0.92)	0.63 (0.12 – 0.88)	0.94 (0.80 – 0.98)	0.78 (0.54 – 0.92)	1.31 (0.92 – 2.22)	1.68 (1.19 – 2.85)	0.57 (0.41 – 0.98)	1.02 (0.81 – 1.42)
	3	1.9 (0.3)$_{a}$	1.8 (0.4)	1.9 (0.4)	0.82 (0.48 – 0.94)	0.79 (0.42 – 0.93)	0.98 (0.94 – 0.99)	0.87 (0.71 – 0.96)	1.05 (0.75 – 1.79)	1.14 (0.81 – 1.94)	0.31 (0.22 – 0.53)	0.79 (0.63 – 1.11)
	4	2.0 (0.3)^{a}	1.9 (0.5)$_{a}$	2.0 (0.3)$_{a}$	0.64 (0.13 – 0.88)	0.81 (0.46 – 0.94)	0.88 (0.62 – 0.96)	0.77 (0.53 – 0.92)	1.65 (1.17 – 2.80)	1.09 (0.77 – 1.85)	0.84 (0.60 – 1.43)	1.03 (0.82 – 1.45)
	5	2.0 (0.3)$_{ab}$	2.0 (0.4)$_{a}$	2.0 (0.4)$_{ab}$	0.65 (0.15 – 0.89)	0.56 (0.02 – 0.85)	0.96 (0.86 – 0.99)	0.75 (0.50 – 0.91)	1.60 (1.13 – 2.72)	1.92 (1.36 – 3.25)	0.47 (0.33 – 0.79)	1.07 (0.85 – 1.50)

ACC = acceleration Tx = thorax longitudinal direction, Ty = thorax medio-lateral, Tz = thorax anterior-posterior, Sn = session; SD = standard deviation; ICC= intraclass correlation coefficient; CI = confidence interval; TE (stzd) = standardized typical error; LL = lower limit; UL = upper limit.

† < 0.05 vs. Sn. 1; ‡ < 0.05 vs. Sn. 2. a < 0.05 vs interval 1; b < 0.05 vs. interval 2; c < 0.05 vs. interval 3

Table S12. Reliability of the LyE for pelvis acceleration

ACC	Interval	Mean (SD)			ICC$_{(3,1)}$ (95%CI)			ICC$_{(3,1)}$ mean (95%CI)	TE (stzd) (LL/UL)			TE (stzd) mean (LL/UL)
		Sn. 1	Sn. 2	Sn. 3	2-1	3-1	3-2		2-1	3-1	3-2	
**Px**	1	1.4 (0.3)	1.3 (0.2)	1.2 (0.2)†	0.01 (-0.55-0.56)	0.07 (-0.50 -0.60)	0.50 (-0.07-0.83)	0.13 (-0.14-0.53)	21.79 (15.44-37.00)	7.77 (5.51-13.20)	2.16 (1.53 – 3.67)	1.87 (1.49 – 2.63)
	2	1.3 (0.2)	1.2 (0.2)	1.2 (0.1)†	0.15 (-0.44-0.65)	0.22 (-0.38-0.69)	0.64 (0.13-0.88)	0.27 (-0.04-0.64)	5.07 (3.59 – 8.60)	4.05 (2.87 – 6.88)	1.64 (1.17 – 2.79)	1.74 (1.39 – 2.44)
	3	1.3 (0.3)	1.2 (0.2)	1.2 (0.2)	0.02 (-0.54-0.57)	0.40 (-0.20-0.78)	0.68 (0.20 – 0.90)	0.32 (0.00 – 0.68)	13.34 (9.45-22.66)	2.64 (1.87 – 4.47)	1.51 (1.87 – 4.47)	1.69 (1.34 – 2.36)
	4	1.3 (0.2)	1.2 (0.2)	1.2 (0.2)	0.05 (-0.52-0.59)	0.50 (-0.07-0.82)	0.73 (0.29 – 0.91)	0.39 (0.06 – 0.73)	8.96 (6.35-15.22)	2.17 (1.54 – 3.68)	1.35 (0.96 – 2.30)	1.61 (1.28 – 2.25)
	5	1.3 (0.2)	1.2 (0.2)	1.2 (0.2)	0.12 (-0.46-0.63)	0.59 (0.05 – 0.86)	0.62 (0.10 – 0.87)	0.46 (0.13 – 0.77)	5.74 (4.07 – 9.75)	1.82 (1.29 – 3.10)	1.72 (1.22 – 2.92)	1.52 (1.21 – 2.13)
**Py**	1	1.4 (0.2)	1.4 (0.2)	1.4 (0.2)	0.00 (-0.55-0.55)	0.06 (-0.51-0.59)	0.63 (0.12 – 0.88)	0.16 (-0.12-0.56)	43.14 (30.56-73.66)	8.63 (6.11-14.65)	1.68 (1.19 – 2.85)	1.85 (1.47 – 2.59)
	2	1.4 (0.2)	1.3 (0.2)a	1.3 (0.1)	0.13 (-0.45 –0.64)	0.18 (-0.41-0.67)	0.79 (0.41 – 0.93)	0.31 (-0.01-0.67)	5.45 (3.86 – 9.26)	4.52 (3.20 – 7.67)	1.16 (0.82 – 1.97)	1.70 (1.35 – 2.38)
	3	1.4 (0.2)	1.4 (0.2)	1.3 (0.1)	0.37 (-0.23-0.77)	0.35 (-0.25-0.76)	0.78 (0.41-0.93)	0.46 (0.13-0.77)	2.82 (2.00-4.78)	2.91 (2.06-4.93)	1.16 (0.82-1.97)	1.52 (1.21 – 2.12)
	4	1.4 (0.2)	1.4 (0.2)	1.4 (0.1)	0.44 (-0.15-0.80)	0.44 (-0.15-0.80)	0.72 (0.28 – 0.91)	0.52 (0.19 – 0.80)	2.44 (1.73 – 4.14)	2.44 (1.73 – 4.14)	1.36 (0.97 – 2.32)	1.45 (1.15 – 2.03)
	5	1.4 (0.2)	1.4 (0.2)	1.4 (0.2)	0.61 (0.09 – 0.87)	0.57 (0.03 – 0.86)	0.71 (0.26 – 0.91)	0.63 (0.33 – 0.86)	1.73 (1.23 – 2.94)	1.88 (1.33 – 3.19)	1.40 (0.99 – 2.38)	1.28 (1.02 – 1.79)
**Pz**	1	1.9 (0.3)	1.8 (0.5)	1.7 (0.3)†	0.53 (-0.04-0.84)	0.49 (-0.08-0.82)	0.69 (0.21 – 0.90)	0.58 (0.26 – 0.83)	2.06 (1.46 – 3.50)	2.20 (1.56 – 3.74)	1.49 (1.06 – 2.53)	1.36 (1.08 – 1.90)
	2	1.8 (0.3)	1.7 (0.4)	1.7 (0.3)	0.77 (0.37 – 0.93)	0.71 (0.26 – 0.91)	0.65 (0.16 – 0.89)	0.71 (0.44 – 0.89)	1.22 (0.87 – 2.08)	1.41 (1.00 – 2.39)	1.60 (1.13 – 2.71)	1.15 (0.91 – 1.61)
	3	1,8 (0.3)	1.7 (0.4)	1.7 (0.3)	0.82 (0.49 – 0.95)	0.79 (0.42 – 0.93)	0.62 (0.10 – 0.87)	0.74 (0.49 – 0.91)	1.04 (0.73 – 1.76)	1.14 (0.81 – 1.94)	1.72 (1.22 – 2.93)	1.09 (0.86 – 1.52)
	4	1.8 (0.3)	1.7 (0.4)	1.7 (0.3)	0.78 (0.39 – 0.93)	0.83 (0.51 – 0.95)	0.60 (0.08 – 0.87)	0.73 (0.47 – 0.90)	1.19 (0.84 – 2.01)	1.00 (0.71 – 1.71)	1.77 (1.25 – 3.00)	1.11 (0.88 – 1.55)
	5	1.8 (0.3)	1.8 (0.3)	1.7 (0.3)	0.81 (0.47 – 0.94)	0.89 (0.67 – 0.97)	0.84 (0.53 – 0.95)	0.84 (0.66 – 0.95)	1.07 (0.76 – 1.82)	0.77 (0.55 – 1.31)	0.98 (0.70 – 1.67)	0.86 (0.69 – 1.21)

ACC = acceleration Px = pelvis longitudinal direction, Py = pelvis medio-lateral, Pz = pelvis anterior-posterior, Sn = session; SD = standard deviation; ICC= intraclass correlation coefficient; CI = confidence interval; TE (stzd) = standardized typical error; LL = lower limit; UL = upper limit. † < 0.05 vs. Sn. 1; ‡ < 0.05 vs. Sn. 2. a < 0.05 vs interval 2

Table S13. Reliability of the LyE for the left shank acceleration

ACC	Interval	Mean (SD)			ICC$_{(3,1)}$ (95%CI)			ICC$_{(3,1)}$ mean (95%CI)	TE (stzd) (LL/UL)			TE (stzd) mean (LL/UL)
		**Sn. 1**	**Sn. 2**	**Sn. 3**	**2-1**	**3-1**	**3-2**		**2-1**	**3-1**	**3-2**	
**LSx**	1	2.7 (0.2)	2.8 (0.3)	2.8 (0.2)	0.58 (0.05-0.86)	0.70 (0.24-0.90)	0.82 (0.50 - 0.95)	0.70 (0.42 - 0.89)	1.84 (1.30 - 3.13)	1.45 (1.03 - 2.46)	1.03 (0.73 – 1.74)	1.17 (0.93 – 1.64)
	2	2.7 (0.3)	2.7 (0.3)	2.7 (0.3)	0.61 (0.09-0.87)	0.73 (0.30-0.92)	0.83 (0.51-0.95)	0.72 (0.45 – 0.90)	1.75 (1.24 – 2.97)	1.33 (0.94 – 2.25)	1.02 (0.73 – 1.73)	1.13 (0.90 – 1.58)
	3	2.7 (0.3)	2.8 (0.3)†	2.7 (0.3)	0.69 (0.23-0.90)	0.72 (0.29-0.91)	0.92 (0.74-0.98)	0.78 (0.54-0.92)	1.46 (1.03-2.48)	1.36 (0.96-2.31)	0.68 (0.48-1.16)	1.02 (0.81-1.43)
	4	2.7 (0.3)	2.8 (0.3)†	2.8 (0.3)†	0.59 (0.06-0.86)	0.76 (0.36-0.92)	0.89 (0.66-0.97)	0.74 (0.49 – 0.91)	1.81 (1.28 – 3.08)	1.24 (0.88 – 2.11)	0.79 (0.56 – 1.33)	1.09 (0.87 – 1.52)
	5	2.7 (0.2)	2.7 (0.3)	2.8 (0.3)	0.50 (-0.08-0.82)	0.74 (0.32-0.92)	0.88 (0.63-0.96)	0.72 (0.45 – 0.90)	2.18 (1.54 – 3.70)	1.31 (0.93 – 2.22)	0.84 (0.60 – 1.43)	1.14 (0.90 – 1.59)
**LSy**	1	3.1 (0.2)	3.2 (0.2)	3.1 (0.2)	0.35 (-0.25-0.76)	0.39 (-0.21-0.78)	0.91 (0.72-0.97)	0.55 (0.23 – 0.82)	2.91 (2.06 – 4.94)	2.70 (1.91 – 4.58)	0.71 (0.50 – 1.20)	1.40 (1.12 – 1.96)
	2	3.0 (0.2)	3.1 (0.3)	3.1 (0.2)	0.50 (-0.08-0.82)	0.48 (-0.10-0.82)	0.88 (0.64-0.96)	0.63 (0.32 – 0.86)	2.19 (1.55 – 3.71)	2.25 (1.59 – 3.81)	0.82 (0.58 – 1.39)	1.29 (1.02 – 1.80)
	3	3.0 (0.3)	3.1 (0.2)†	3.1 (0.2)	0.68 (0.20-0.89)	0.55 (-0.01-0.84)	0.89 (0.67-0.97)	0.70 (0.42-0.89)	1.52 (1.08-2.58)	1.98 (1.40-3.36)	0.78 (0.55-1.32)	1.17 (0.93-1.64)
	4	3.0 (0.3)	3.1 (0.2)†	3.1 (0.2)†	0.73 (0.30-0.91)	0.59 (0.06-0.86)	0.85 (0.57-0.96)	0.71 (0.44 – 0.89)	1.34 (0.95-2.28)	1.81 (1.28 – 3.07)	0.92 (0.65 – 1.57)	1.15 (0.91-1.60)
	5	3.0 (0.3)	3.1 (0.2)†	3.1 (0.2)†	0.70 (0.24-0.90)	0.64 (0.14-0.88)	0.86 (0.58-0.96)	0.72 (0.45 – 0.90)	1.43 (1.01 – 2.43)	1.63 (1.15 – 2.76)	0.91 (0.64 – 1.55)	1.13 (0.90 – 1.58)
**LSz**	1	3.1 (0.3)	3.3 (0.2)	3.1 (0.3)	0.24 (-0.36-0.70)	0.53 (-0.03-0.84)	0.78 (0.61-0.88)	0.60 (0.44-0.75)	2.05 (1.67-2.66)	1.98 (1.61-2.56)	1.10 (0.90-1.42)	1.28 (1.12-1.51)
	2	3.1 (0.3)	3.2 (0.2)†	3.1 (0.3)‡	0.11 (-0.47-0.63)	0.49 (-0.08-0.82)	0.52 (-0.04-0.83)	0.39 (0.06 – 0.73)	6.01 (4.25–10.20)	2.20 (1.56 – 3.74)	2.08 (1.47 – 3.53)	1.60 (1.28 – 2.25)
	3	3.1 (0.3)	3.2 (0.2)†	3.1 (0.3)	0.22 (-0.38-0.69)	0.57 (0.03-0.85)	0.27 (-0.33-0.72)	0.38 (0.06 – 0.72)	4.05 (2.87 - 6.88)	1.88 (1.33 – 3.19)	3.49 (2.47 – 5.93)	1.61 (1.28 – 2.26)
	4	3.1 (0.3)	3.3 (0.2)†	3.1 (0.3)‡	0.36 (-0.24-0.76)	0.55 (0.00-0.85)	0.24 (-0.36-0.70)	0.40 (0.07 – 0.73)	2.86 (2.03 – 4.86)	1.97 (1.39 – 3.34)	3.83 (2.72 – 6.51)	1.60 (1.27 – 2.23)
	5	3.1 (0.2)	3.3 (0.2)†	3.2 (0.3)‡	0.26 (-0.34-0.71)	0.35 (-0.25-0.76)	0.62 (0.10-0.87)	0.44 (0.11 – 0.76)	3.61 (2.56 – 6.13)	1.72 (1.22 – 2.92)	2.91 (2.06 – 4.95)	1.54 (1.23 – 2.16)
**RSx**	1	2.7 (0.3)	2.8 (0.3)	2.7 (0.3)	0.61 (0.08-0.87)	0.76 (0.36-0.92)	0.68 (0.20-0.89)	0.67 (0.38 – 0.88)	1.75 (1.24 – 2.98)	1.24 (0.88-2.10)	1.52 (1.08 – 2.58)	1.21 (0.97 – 1.70)
	2	2.6 (0.3)	2.8 (0.3)†	2.7 (0.3)	0.82 (0.49-0.94)	0.86 (0.59-0.96)	0.77 (0.37-0.93)	0.81 (0.61-0.94)	1.04 (0.74 – 1.77)	0.89 (0.63-1.51)	1.22 (0.87 – 2.08)	0.94 (0.74 – 1.31)
	3	2.6 (0.3)	2.8 (0.4)†	2.7 (0.3)	0.82 (0.48-0.94)	0.63 (0.12-0.88)	0.72 (0.28-0.91)	0.79 (0.56-0.93)	1.06 (0.75-1.80)	1.18 (0.83-2.00)	1.37 (0.97 – 2.33)	0.99 (0.79 – 1.39)

ACC	Interval	Mean (SD)			$ICC_{(3,1)}$ (95%CI)			$ICC_{(3,1)}$ mean (95%CI)	TE (stzd) (LL/UL)			TE (stzd) mean (LL/UL)
		**Sn. 1**	**Sn. 2**	**Sn. 3**	**2-1**	**3-1**	**3-2**		**2-1**	**3-1**	**3-2**	
	4	2.6<br>(0.3)	2.8<br>(0.4)†	2.7<br>(0.2)	0.74<br>(0.32-0.92)	0.81<br>(0.46-0.94)	0.64<br>(0.13-0.88)	0.73<br>(0.46 – 0.90)	1.30<br>(0.92 – 2.21)	1.08<br>(0.77 – 1.84)	1.65<br>(1.17 – 2.81)	1.12<br>(0.89 – 1.57)
	5	2.7<br>(0.3)	2.8<br>(0.4)	2.8<br>(0.3)	0.75<br>(0.35-0.92)	0.80<br>(0.45-0.94)	0.68<br>(0.20-0.90)	0.74<br>(0.49 – 0.91)	1.26<br>(0.89 – 2.14)	1.10<br>(0.78 – 1.86)	1.51<br>(1.07 – 2.56)	1.09<br>(0.86 – 1.52)
RSy	1	3.1<br>(0.2)	3.1<br>(0.4)	3.1<br>(0.1)	0.28<br>(-0.32-0.72)	0.54<br>(-0.02-0.84)	0.41<br>(-0.18-0.79)	0.38<br>(0.05-0.72)	3.43<br>(2.43-5.83)	2.01<br>(1.42 – 3.41)	2.57<br>(1.82-4.37)	1.62<br>(1.29-2.27)
	2	3.0<br>(0;2)	3.1<br>(0.3)	3.2<br>(0.2)	0.54<br>(-0.01-0.84)	0.58<br>(0.05-0.60)	0.60<br>(0.07-0.86)	0.57<br>(0.25-0.83)	1.99<br>(1.41 – 3.38)	1.84<br>(1.30-3.12)	1.79<br>(1.27 – 3.05)	1.37<br>(1.09 – 1.92)
	3	3.0<br>(0.3)	3.0<br>(0.4)	3.2<br>(0.2)	0.63<br>(0.12-0.88)	0.56<br>(0.01-0.85)	0.60<br>(0.07-0.86)	0.60<br>(0.29 – 0.84)	1.68<br>(1.19 – 2.85)	1.94<br>(1.37 - 3.29)	1.79<br>(1.27 - 3.04)	1.33<br>(1.06 – 1.86)
	4	3.0<br>(0.3)	3.0<br>(0.4)	3.2<br>(0.2)†	0.68<br>(0.20-0.90)	0.63<br>(0.11-0.88)	0.52<br>(-0.05-0.83)	0.61<br>(0.30-0.85)	1.51<br>(1.07 – 2.56)	1.69<br>(1.20 – 2.87)	2.10<br>(1.48-3.56)	1.31<br>(1.04 – 1.84)
	5	3.0<br>(0.3)	3.1<br>(0.4)	3.2<br>(0.2)†	0.67<br>(0.19-0.89)	0.71<br>(0.26-0.91)	0.66<br>(0.16-0.89)	0.67<br>(0.39-0.88)	1.54<br>(1.09-2.61)	1.41<br>(1.00-2.40)	1.59<br>(1.12-2.70)	1.21<br>(0.96 – 1.70)
RSz	1	3.0<br>(0.3)	3.1<br>(0.3)	3.0<br>(0.3)	0.01<br>(-0.55-0.56)	**-0.06**<br>**(-0.60-0.51)**	0.82<br>(0.49-0.94)	0.27<br>(-0.04-0.64)	29.35<br>(20.79-49.83)	**0.59**<br>**(0.42-1.00)**	0.66<br>(0.54-0.86)	1.74<br>(1.39 - 2.44)
	2	3.0<br>(0.3)	3.1<br>(0.3)	3.0<br>(0.3)	0.05<br>(-0.51-0.59)	0.03<br>(-0.53-0.57)	0.78<br>(0.39-0.93)	0.30<br>(-0.01-0.67)	8.89<br>(6.30-15.09)	11.74<br>(8.32-19.93)	1.19<br>(0.84-2.01)	1.70<br>(1.36-2.39)
	3	3.0<br>(0.3)	3.1<br>(0.3)	3.1<br>(0.2)	0.03<br>(-0.53-0.57)	0.04<br>(-0.52-0.58)	0.76<br>(-0.52-0.58)	0.28<br>(-0.04-0.65)	12.92<br>(9.15-21.93)	9.95<br>(7.05-16.90)	1.25<br>(0.89-2.13)	1.73<br>(1.38-2.43)
	4	3.1<br>(0.3)	3.1<br>(0.3)	3.1<br>(0.2)	**-0.03**<br>**(-0.58-0.53)**	0.01<br>(-0.54-0.56)	0.57<br>(0.02-0.85)	0.15<br>(-0.13-0.55)	**0.58**<br>**(0.41-0.98)**	19.49<br>(13.81-33.09)	1.91<br>(1.35-3.24)	1.86<br>(1.48-2.60)
	5	3.1<br>(0.3)	3.1<br>(0.3)	3.1<br>(0.2)	0.07<br>(-0.50-0.60)	0.07<br>(-0.50-0.60)	0.52<br>(-0.04-0.84)	0.20<br>(-0.10-0.59)	7.77<br>(5.51-13.20)	7.67<br>(5.43-13.01)	2.07<br>(1.46-3.51)	1.81<br>(1.44-2.54)

ACC = acceleration, LSx = left shank longitudinal direction, LSy = left shank medio-lateral, LSz = left shank anterior-posterior, RSx = right shank longitudinal, RSy = right shank acceleration direction, RSz = right shank anterior-posterior, Sn = session; SD = standard deviation; ICC = intraclass correlation coefficient; CI = confidence interval; TE (stzd) = standardized typical error; LL = lower limit; UL = upper limit.

† < 0.05 vs. Sn. 1; ‡ < 0.05 vs. Sn. 2.

Note: Bolded is when raw data was used

Table S14. Reliability of the LyE for neck angular kinematics

Kin	Interval	Mean (SD)			$ICC_{(3,1)}$ (95%CI)			$ICC_{(3,1)}$ mean (95%CI)	TE (stzd) (LL/UL)			TE (stzd) mean (LL/UL)
		**Sn. 1**	**Sn. 2**	**Sn. 3**	**2-1**	**3-1**	**3-2**		**2-1**	**3-1**	**3-2**	
**Nx**	1	2.5 (0.4)	2.5 (0.4)	2.5 (0.5)	0.19 (-0.40-0.67)	0.37 (-0.23-0.77)	0.64 (-0.23-0.77)	0.41 (0.09 – 0.74)	4.34 (3.07 – 7.37)	2.81 (1.99 – 4.78)	1.64 (1.16 – 2.79)	1.58 (1.26 – 2.21)
	2	2.5 (0.5)	2.5 (0.5)	2.4 (0.4)	0.31 (-0.29-0.74)	0.22 (-0.38-0.69)	0.84 (0.53 – 0.95)	0.46 (0.13 – 0.77)	3.18 (2.25 – 5.40)	4.00 (2.83 – 6.79)	0.98 (0.70 – 1.67)	1.52 (1.21 – 2.13)
	3	2.4 (0.5)	2.6 (0.4)	2.4 (0.7)	0.39 (-0.21-0.78)	**-0.09 (-0.61-0.49)***	0.53 (-0.03-0.84)	0.25 (-0.05-0.63)	2.69 (1.91 – 4.57)	**1.19 (0.84-2.02)***	2.05 (1.45 – 3.47)	1.76 (1.40 – 2.46)
	4	2.6 (0.3)	2.5 (0.4)	2.6 (0.4)	0.99 (0.98 – 1.00)	0.44 (-0.22-0.80)	1.00 (0.99 – 1.00)	0.99 (0.98 – 1.00)	0.18 (0.12 – 0.31)	0.14 (0.10 – 0.24)	2.44 (1.70 – 4.28)	0.18 (0.15 – 0.25)
	5	2.7 (0.4)$_c$	2.7 (0.4)	2.6 (0.5)$_c$	0.57 (0.02 – 0.85)	0.51 (-0.06-0.83)	0.92 (0.75 – 0.98)	0.68 (0.40 – 0.88)	1.90 (1.35 – 3.23)	2.12 (1.50 – 3.59)	0.66 (0.47 – 1.13)	1.20 (0.95 – 1.68)
**Ny**	1	1.8 (0.9)	2.0 (0.8)	2.1 (0.9)	**-0.08 (-0.60-0.50)***	0.54 (-0.01-0.84)	0.01 (-0.54-0.56)	0.18 (-0.11-0.57)	**1.79 (1.27-3.04)***	1.99 (1.41 – 3.39)	18.99 (13.38-32.07)	0.87 (0.76-1.03)
	2	2.0 (0.8)	1.8 (0.9)	1.9 (0.8)	0.65 (0.16 – 0.89)	0.87 (0.61 – 0.96)	0.79 (0.42 – 0.93)	0.77 (0.53 – 0.92)	1.60 (1.13 – 2.71)	0.87 (0.61 – 1.47)	1.14 (0.81 – 1.94)	1.04 (0.83 – 1.46)
	3	2.1 (0.8)	1.9 (0.9)	2.1 (0.7)	0.76 (0.35 – 0.92)	0.90 (0.68 – 0.97)	0.87 (0.60 – 0.96)	0.84 (0.65 – 0.94)	1.25 (0.89 – 2.12)	0.76 (0.54 – 1.30)	0.88 (0.62 – 1.49)	0.88 (0.70 – 1.23)
	4	2.2 (0.8)	2.0 (0.8)	2.1 (0.8)	0.78 (0.41 – 0.93)	0.95 (0.83 – 0.99)	0.87 (0.61 – 0.96)	0.87 (0.71 – 0.96)	1.17 (0.83 – 1.98)	0.52 (0.37 – 0.88)	0.86 (0.61 – 1.46)	0.79 (0.63 – 1.11)
	5	2.3 (0.9)$_a$	2.2 (0.6)$_b$	2.2 (0.8)	0.30 (-0.30-0.73)	0.95 (0.83 – 0.98)	0.49 (-0.08-0.82)	0.64 (0.34 – 0.86)	3.27 (2.31 – 5.55)	0.53 (0.38 – 0.90)	2.21 (1.56 – 3.74)	1.27 (1.01 – 1.78)
**Nz**	1	0.3 (0.6)	0.1 (1.5)	0.2 (0.6)	0.51 (-0.06-0.83)	0.34 (-0.26-0.75)	0.49 (-0.09-0.82)	0.43 (0.10 – 0.75)	2.14 (1.52 – 3.64)	2.22 (1.57 – 3.76)	2.96 (2.10 – 5.03)	1.55 (1.24 – 2.18)
	2	0.6 (1.0)	0.8 (1.2)$_a$	0.2 (0.6)$_{‡}$	0.85 (0.57 – 0.96)	0.65 (0.15 – 0.88)	0.46 (-0.13 –0.81)	0.68 (0.39 – 0.88)	0.92 (0.65 – 1.57)	1.62 (1.15 – 2.75)	2.36 (1.67 – 4.00)	1.20 (0.96 – 1.68)
	3	0.9 (1.2)$_a$	0.6 (1.2)$_a$	0.4 (0.9)	0.83 (0.51 – 0.95)	0.55 (-0.01-0.84)	0.73 (0.30 – 0.91)	0.72 (0.44 – 0.90)	1.01 (0.72 – 1.72)	1.98 (1.40 – 3.37)	1.34 (0.95 – 2.28)	1.14 (0.91 – 1.60)
	4	0.9 (1.2)$_a$	0.8 (1.2)$_a$	0.5 (0.9)	0.69 (0.22 – 0.90)	0.79 (0.41 – 0.93)	0.58 (0.05 – 0.86)	0.69 (0.41 – 0.88)	1.47 (1.04 – 2.50)	1.15 (0.82 – 1.96)	1.84 (1.30 – 3.12)	1.19 (0.95 – 1.66)
	5	0.5 (1.1)	0.9 (1.2)$_a$	0.5 (0.9)	0.87 (0.61 – 0.96)	0.91 (0.72 – 0.97)	0.84 (0.54 – 0.95)	0.87 (0.71 – 0.96)	0.86 (0.61 – 1.46)	0.71 (0.50 – 1.20)	0.97 (0.68 – 1.64)	0.79 (0.63 – 1.10)

Kin = Kinematics, Nx = neck flexion, Ny = neck lateral flexion, Nz = neck rotation, Sn = session; SD = standard deviation; ICC = intraclass correlation coefficient; CI = confidence interval; TE (stzd) = standardized typical error; LL = lower limit; UL = upper limit.

† < 0.05 vs. Sn. 1; ‡ < 0.05 vs. Sn. 2. a < 0.05 vs interval 1; b < 0.05 vs. interval 2; c < 0.05 vs. interval 3

Note: bolded* = raw data used to calculate typical error

Table S15. Reliability of the LyE for thorax angular kinematics

Kin	Interval	Mean (SD)			ICC$_{(3,1)}$ (95%CI)			ICC$_{(3,1)}$ mean (95%CI)	TE (stzd) (LL/UL)			TE (stzd) mean (LL/UL)
		Sn. 1	Sn. 2	Sn. 3	2-1	3-1	3-2		2-1	3-1	3-2	
Tx	1	2.6 (0.5)	2.5 (0.9)	2.8 (0.2)	0.33 (-0.27-0.75)	0.07 (-0.50-0.60)	0.19 (-0.40-0.67)	0.24 (-0.06-0.62)	1.40 (1.14-1.81)	1.40 (1.14-1.81)	0.97 (0.79-1.25)	1.05 (0.92-1.25)
	2	2.7 (0.6)	2.3 (0.8)	2.4 (0.8)	0.53 (-0.03-0.84)	0.12 (-0.47-0.63)	0.32 (-0.28-0.74)	0.33 (0.01 – 0.69)	2.05 (1.45 – 3.48)	5.84 (4.14 – 9.92)	3.10 (2.19 – 5.26)	1.67 (1.33 – 2.34)
	3	2.7 (0.6)	2.4 (0.8)	2.5 (0.9)	0.42 (-0.17-0.79)	0.16 (-0.43-0.66)	0.10 (-0.48-0.62)	0.21 (-0.08-0.60)	2.54 (1.80 – 4.31)	4.85 (3.43 – 8.23)	6.37 (4.51-10.81)	1.80 (1.43 – 2.52)
	4	2.7 (0.5)	2.5 (0.8)	2.6 (0.6)	0.31 (-0.29-0.74)	0.09 (-0.48-0.61)	0.27 (-0.34-0.71)	0.25 (-0.06-0.63)	3.19 (2.26 – 5.42)	6.59 (4.67 – 11.20)	3.55 (2.51 – 6.03)	1.76 (1.40 – 2.47)
	5	2.9 (0.5)	2.6 (0.7)	2.7 (0.4)	0.10 (-0.48-0.62)	**-0.22 (-0.69-0.38)***	0.11 (-0.47-0.62)	0.03 (-0.21-0.43)	6.31 (4.47 – 10.72)	**1.02 (0.72-1.74)***	6.12 (4.33-10.39)	1.97 (1.57 – 2.77)
Ty	1	2.5 (0.4)	2.9 (0.3)†	2.9 (0.3)	**-0.08 (-0.61-0.49)***	0.52 (-0.05-0.83)	**-0.26 (-0.71-0.34)***	0.11 (-0.16-0.51)	**0.68 (0.48-1.16)***	2.10 (1.49 – 3.57)	**0.65 (0.46-1.10)***	1.90 (1.51 – 2.66)
	2	2.6 (0.5)	2.8 (0.3)	2.8 (0.4)	0.31 (-0.30-0.74)	0.30 (-0.30-0.73)	0.39 (-0.20-0.78)	0.33 (0.01 – 0.69)	3.22 (2.28 – 5.47)	3.26 (2.31 – 5.53)	2.68 (1.90 – 4.55)	1.68 (1.34 – 2.35)
	3	2.7 (0.4)	2.9 (0.3)	2.9 (0.4)	0.37 (-0.23-0.76)	0.60 (0.08 – 0.87)	0.51 (-0.06-0.83)	0.50 (0.17 – 0.79)	2.83 (2.01 – 4.81)	1.77 (1.25 – 3.01)	2.14 (1.51 – 3.63)	1.47 (1.17 – 2.06)
	4	2.7 (0.4)	2.9 (0.3)†	2.9 (0.3)†	0.25 (-0.35-0.70)	0.49 (-0.08-0.82)	0.74 (0.32 – 0.92)	0.49 (0.17 – 0.79)	3.73 (2.64 – 6.33)	2.19 (1.55 – 3.72)	1.30 (0.92 – 2.20)	1.48 (1.18 – 2.07)
	5	2.8 (0.4)a	3.0 (0.3)b	2.9 (0.4)	0.57 (0.03 – 0.85)	0.45 (-0.14-0.80)	0.61 (0.08 – 0.87)	0.54 (0.21 – 0.81)	1.89 (1.34 – 3.21)	2.39 (1.69 – 4.06)	1.75 (1.24 – 2.97)	1.42 (1.13 – 1.99)
Tz	1	-0.1 (0.0)	-0.1 (0.0)	-0.1 (0.0)	0.62 (0.11 – 0.87)	0.12 (-0.46-0.63)	0.17 (-0.43-0.66)	0.32 (0.00 – 0.68)	1.70 (1.21 – 2.89)	5.71 (4.04 – 9.69)	4.77 (3.38 – 8.10)	1.68 (1.34 – 2.36)
	2	-0.1 (0.1)	0.2 (0.8)	-0.1 (0.0)	**-0.06 (-0.60-0.51)***	0.58 (0.04 – 0.86)	**0.00 (-0.55-0.55)***	-0.03 (-0.24-0.36)	**1.15 (0.81-1.95)***	1.85 (1.31 – 3.15)	**1.85 (1.31-3.15)***	2.03 (1.61 – 2.84)
	3	-0.1 (0.0)	0.2 (0.8)	-0.1 (0.0)	**-0.01 (-0.56-0.55)***	0.26 (-0.35-0.71)	**-0.03 (0.58-0.53)***	-0.02 (-0.24-0.37)	**1.16 (0.82-1.97)***	3.63 (2.57 – 6.17)	**1.17 (0.83-1.99)***	2.02 (1.61 – 2.83)
	4	-0.1 (0.1)	0.2 (0.8)	-0.1 (0.0)	**-0.08 (-0.60-0.50)***	**-0.05 (-0.59-0.52)***	0.02 (-0.54-0.57)	-0.03 (-0.24 –0.36)	**1.24 (0.88-2.11)***	**0.09 (0.07-0.16)***	13.66 (9.68 – 23.19)	2.02 (1.61 – 2.83)
	5	-0.1 (0.0)	0.2 (0.9)	-0.1 (0.0)	0.01 (-0.55-0.56)	**-0.03 (-0.57-0.53)***	**-0.04 (-0.58-0.53)***	-0.01 (-0.23-0.38)	20.94 (14.84-35.56)	**0.08 (0.05-0.13)***	**1.29 (0.91-2.19)***	2.01 (1.60 – 2.82)

Kin = Kinematics, Tx = spinal flexion, Ty = spinal lateral flexion, Tz = thorax rotation, Sn = session; SD = standard deviation; ICC = intraclass correlation coefficient; CI = confidence interval; TE (stzd) = standardized typical error; LL = lower limit; UL = upper limit.

† < 0.05 vs. Sn. 1; ‡ < 0.05 vs. Sn. 2. a < 0.05 vs interval 1; b < 0.05 vs. interval 2

Note: bolded* = raw data used to calculate typical error

Table S16. Reliability of the LyE for pelvis angular kinematics

Kin	Interval	Mean (SD)			$ICC_{(3,1)}$ (95%CI)			$ICC_{(3,1)}$ mean (95%CI)	TE (stzd) (LL/UL)			TE (stzd) mean (LL/UL)
		**Sn. 1**	**Sn. 2**	**Sn. 3**	**2-1**	**3-1**	**3-2**		**2-1**	**3-1**	**3-2**	
Px	1	-0.1 (0.0)	-0.1 (0.0)	-0.1 (0.0)	0.06 (-0.51-0.59)	0.39 (-0.21-0.77)	0.63 (0.12 – 0.88)	0.34 (0.02 – 0.70)	8.25 (5.85-14.01)	2.71 (1.92 – 4.60)	1.67 (1.18 – 2.83)	1.66 (1.32 – 2.32)
	2	-0.1 (0.0)	-0.1 (0.0)	-0.1 (0.0)	0.72 (0.27 – 0.91)	0.64 (0.14 – 0.88)	0.44 (-0.15–0.80)	0.61 (0.30 – 0.85)	1.39 (0.98 – 2.36)	1.64 (1.16 – 2.78)	2.46 (1.74 – 4.17)	1.32 (1.05 – 1.85)
	3	-0.1 (0.0)$_{a}$	-0.1 (0.0)	-0.1 (0.0)$_{\dagger}$	0.34 (-0.26-0.75)	0.56 (0.01 – 0.85)	0.26 (-0.35-0.71)	0.41 (0.08 – 0.74)	2.96 (2.10 – 5.03)	1.93 (1.36 – 3.27)	3.65 (2.59 – 6.20)	1.59 (1.26 – 2.22)
	4	-0.1 (0.0)	-0.1 (0.0)	-0.1 (0.0)$_{b}$	0.42 (-0.17-0.79)	0.43 (-0.16-0.79)	0.53 (-0.03-0.84)	0.47 (0.14 – 0.77)	2.53 (1.79 – 4.29)	2.49 (1.77 – 4.23)	2.05 (1.45 – 3.48)	1.51 (1.20 – 2.12)
	5	-0.1 (0.0)	-0.1 (0.0)	-0.1 (0.0)	0.51 (-0.05-0.83)	0.18 (-0.42-0.66)	0.60 (0.07 – 0.86)	0.44 (0.11 – 0.76)	2.11 (1.49 – 3.58)	4.61 (3.26 – 7.82)	1.80 (1.27 – 3.05)	1.54 (1.23 – 2.16)
Py	1	-0.1 (0.1)	-0.1 (0.1)$_{\dagger}$	-0.1 (0.1)$_{\dagger}$	0.31 (-0.29-0.74)	0.24 (-0.36-0.70)	0.19 (-0.41-0.67)	0.25 (-0.06-0.63)	3.17 (2.25 – 5.38)	3.75 (2.66 – 6.37)	4.43 (3.14 – 7.53)	1.76 (1.40 – 2.47)
	2	-0.1 (0.0)	-0.1 (0.0)	-0.1 (0.1)	0.55 (-0.01-0.84)	0.58 (0.05 – 0.86)	0.92 (0.75 – 0.98)	0.70 (0.43 – 0.89)	1.98 (1.40 – 3.37)	1.84 (1.30 – 3.13)	0.66 (0.46 – 1.11)	1.16 (0.92 – 1.63)
	3	-0.1 (0.0)	-0.1 (0.1)	-0.1 (0.1)	0.34 (-0.26-0.75)	0.31 (-0.29-0.74)	0.68 (0.20 – 0.90)	0.46 (0.13 – 0.77)	2.98 (2.11 – 5.05)	3.18 (2.25 – 5.40)	1.51 (1.07 – 2.56)	1.52 (1.21 – 2.13)
	4	-0.1 (0.0)	-0.1 (0.1)	-0.1 (0.0)	0.69 (0.22 – 0.90)	0.24 (-0.35-0.70)	0.24 (-0.36-0.70)	0.41 (0.08 – 0.74)	1.47 (1.04 – 2.50)	3.79 (2.69 – 6.44)	3.77 (2.67 – 6.41)	1.59 (1.26 – 2.22)
	5	-0.1 (0.1)$_{a}$	-0.1 (0.0)	-0.1 (0.0)	0.47 (-0.11-0.81)	0.57 (0.03 – 0.85)	0.46 (-0.12-0.81)	0.51 (0.18 – 0.80)	2.29 (1.63 – 3.90)	1.89 (1.34 – 3.20)	2.32 (1.64 – 3.94)	1.46 (1.16 – 2.05)
Pz	1	-0.1 (0.0)	0.3 (1.0)	-0.1 (0.0)	**0.00 (-0.56-0.55)***	0.02 (-0.54-0.56)	0.21 (-0.39-0.68)	0.10 (-0.16-0.50)	**1.42 (1.00-2.41)***	17.01 (12.05-28.87)	4.13 (2.92 – 7.01)	1.90 (1.52 – 2.67)
	2	-0.1 (0.0)	0.2 (1.0)	-0.1 (0.1)	**-0.01 (-0.56-0.55)***	**-0.05 (-0.58-0.52)**	0.20 (-0.39-0.68)	0.10 (-0.16-0.50)	**1.35 (0.96-2.30)***	**0.14 (0.10-0.24)***	4.22 (2.99-7.17)	1.91 (1.52 – 2.68)
	3	-0.1 (0.0)	0.2 (1.0)	-0.1 (0.0)	**-0.02 (-0.57-0.54)***	**-0.21 (-0.68-0.39)**	0.03 (-0.53-0.57)	0.00 (-0.22-0.40)	**1.45 (1.03-2.46)***	**0.07 (0.05-0.13)***	12.60 (8.93-21.40)	2.00 (1.59 – 2.80)
	4	-0.1 (0.0)	0.2 (1.0)	-0.1 (0.1)	**-0.02 (-0.57-0.54)***	0.02 (-0.54-0.56)	0.03 (-0.53-0.57)	0.00 (-0.22-0.39)	**1.49 (1.05-2.52)***	16.30 (11.54-27.67)	12.89 (9.13-21.88)	2.00 (1.59-2.80)
	5	-0.1 (0.0)	0.3 (1.0)	-0.1 (0.0)	0.00 (-0.55-0.56)	0.36 (-0.24-0.76)	**0.00 (-0.55-0.55)***	0.00 (-0.22-0.39)	31.76 (22.50 -53.93)	2.86 (2.03 – 4.86)	**1.48 (1.05-2.51)***	2.00 (1.59 – 2.80)

Px = pelvic tilt, Py = pelvic obliquity, Pz = pelvic rotation, Sn = session; SD = standard deviation; ICC = intraclass correlation coefficient; CI = confidence interval; TE (stzd) = standardized typical error; LL = lower limit; UL = upper limit.

† < 0.05 vs. Sn. 1; ‡ < 0.05 vs. Sn. 2. a < 0.05 vs interval 1; b < 0.05 vs. interval 2

Note: bolded* = raw data used to calculate typical error

Table S17. Reliability of the LyE for left and right hip kinematics

Kin	Interval	Mean (SD)			$ICC_{(3,1)}$ (95%CI)			$ICC_{(3,1)}$ mean (95%CI)	TE (stzd) (LL/UL)			TE (stzd) mean (LL/UL)
		Sn. 1	Sn. 2	Sn. 3	2-1	3-1	3-2		2-1	3-1	3-2	
LHx	1	2.5<br>(0.4)	2.4<br>(0.6)	2.3<br>(0.6)	0.61<br>(0.09 – 0.87)	0.65<br>(0.16 – 0.89)	0.25<br>(-0.35-0.71)	0.49<br>(0.16 – 0.78)	1.74<br>(1.23 – 2.95)	1.60<br>(1.13 – 2.71)	3.69<br>(2.62 – 6.27)	1.49<br>(1.18 – 2.08)
	2	2.3<br>(0.4)	2.3<br>(0.4)	2.1<br>(0.7)	0.56<br>(0.02 – 0.85)	0.48<br>(-0.10-0.81)	0.17<br>(-0.43-0.66)	0.37<br>(0.05 – 0.72)	1.91<br>(1.35-3.25)	2.27<br>(1.61-3.86)	4.76<br>(3.37 – 8.08)	1.63<br>(1.29 – 2.28)
	3	2.5<br>(0.4)	2.3<br>(0.5)	2.2<br>(0.6)	0.67<br>(0.18 – 0.89)	0.36<br>(-0.24-0.76)	0.26<br>(-0.35-0.71)	0.40<br>(0.07 - 0.73)	1.55<br>(1.10 – 2.64)	2.84<br>(2.01 – 4.82)	3.64<br>(2.58 – 6.18)	1.59<br>(1.27 – 2.23)
	4	2.5<br>(0.4)	2.3<br>(0.5)	2.2<br>(0.4)	0.56<br>(0.01 – 0.85)	0.30<br>(-0.30-0.73)	0.54<br>(-0.03-0.84)	0.48<br>(0.15 – 0.78)	1.93<br>(1.37 – 3.27)	3.28<br>(2.33 – 5.57)	2.03<br>(1.44 – 3.44)	1.50<br>(1.20 – 2.10)
	5	2.5<br>(0.4)	2.2<br>(0.5)	2.3<br>(0.4)	0.68<br>(0.20 – 0.90)	0.75<br>(0.34 – 0.92)	0.79<br>(0.42 – 0.94)	0.74<br>(0.49 – 0.91)	1.51<br>(1.07 – 2.56)	1.27<br>(0.90 – 2.16)	1.14<br>(0.81 – 1.93)	1.09<br>(0.87 – 1.53)
LHy	1	2.7<br>(0.5)	2.6<br>(0.5)	2.6<br>(0.5)	0.72<br>(0.53-0.85)	0.60<br>(0.35-0.77)	0.84<br>(0.71-0.91)	0.71<br>(0.57-0.82)	1.27<br>(1.03-1.64)	1.68<br>(1.37-2.18)	0.91<br>(0.74-1.17)	1.10<br>(0.96-1.30)
	2	2.6<br>(0.5)	2.5<br>(0.5)	2.6<br>(0.4)	0.69<br>(0.22 – 0.90)	0.64<br>(0.13 – 0.88)	0.54<br>(-0.02-0.84)	0.63<br>(0.32 – 0.86)	1.47<br>(1.04 – 2.50)	1.64<br>(1.16 – 2.79)	2.02<br>(1.43 – 3.43)	1.29<br>(1.02 – 1.80)
	3	2.6<br>(0.5)	2.5<br>(0.6)	2.6<br>(0.5)	0.80<br>(0.45 – 0.94)	0.71<br>(0.25 – 0.91)	0.55<br>(0.00 – 0.85)	0.69<br>(0.41 – 0.89)	1.11<br>(0.78 – 1.88)	1.42<br>(1.01 – 2.41)	1.95<br>(1.38 – 3.31)	1.18<br>(0.94 – 1.65)
	4	2.7<br>(0.5)	2.5<br>(0.5)	2.6<br>(0.4)	0.67<br>(0.18)	0.76<br>(0.35 – 0.92)	0.58<br>(0.04 – 0.86)	0.67<br>(0.37 – 0.87)	1.55<br>(1.10 – 2.63)	1.26<br>(0.89 – 2.13)	1.86<br>(1.31 – 3.15)	1.23<br>(0.98 – 1.72)
	5	2.7<br>(0.5)	2.6<br>(0.5)	2.6<br>(0.5)	0.63<br>(0.12 – 0.88)	0.73<br>(0.30 – 0.92)	0.65<br>(0.15 – 0.89)	0.67<br>(0.38 – 0.88)	1.68<br>(1.19 – 2.85)	1.33<br>(0.94 – 2.26)	1.60<br>(1.13 – 2.72)	1.22<br>(0.97 – 1.71)
LHz	1	2.3<br>(0.6)	2.4<br>(0.5)	2.4<br>(0.3)	0.48<br>(-0.10-0.81)	0.25<br>(-0.35-0.71)	0.84<br>(0.52-0.95)	0.50<br>(0.17 – 0.79)	2.27<br>(1.61 – 3.86)	3.71<br>(2.63 – 6.29)	0.99<br>(0.70 – 1.68)	1.47<br>(1.17 – 2.05)
	2	2.2<br>(0.5)	2.2<br>(0.4)	2.3<br>(0.4)	0.47<br>(-0.11-0.81)	0.30<br>(-0.30-0.73)	0.87<br>(0.61 – 0.96)	0.53<br>(0.21 – 0.81)	2.28<br>(1.62 – 3.88)	3.25<br>(2.30 – 5.51)	0.86<br>(0.61 – 1.46)	1.43<br>(1.14 – 2.00)
	3	2.1<br>(0.7)	2.1<br>(0.6)$_a$	2.0<br>(0.6)$_a$	0.78<br>(0.40 – 0.93)	0.58<br>(0.04 – 0.86)	0.64<br>(0.14 – 0.88)	0.67<br>(0.38 – 0.88)	1.17<br>(0.83 – 1.99)	1.85<br>(1.31 – 3.13)	1.64<br>(1.16 – 2.78)	1.22<br>(0.97 – 1.70)
	4	2.2<br>(0.7)	2.1<br>(0.6)	2.3<br>(0.4)$_c$	0.73<br>(0.29 – 0.91)	0.56<br>(0.01 – 0.85)	0.51<br>(-0.06-0.83)	0.62<br>(0.31 – 0.85)	1.35<br>(0.95 – 2.29)	1.93<br>(1.37 – 3.28)	2.13<br>(1.51 – 3.61)	1.30<br>(1.03 – 1.82)
	5	2.3<br>(0.6)	2.2<br>(0.6)	2.4<br>(0.4)$_c$	0.44<br>(-0.14-0.80)	0.80<br>(0.44 – 0.94)	0.39<br>(-0.21-0.78)	0.53<br>(0.20 - 0.81)	2.41<br>(1.71 – 4.10)	1.12<br>(0.79 – 1.90)	2.68<br>(1.90 – 4.55)	1.43<br>(1.14 – 2.01)
RHx	1	2.5<br>(0.6)	2.6<br>(0.9)	2.8<br>(0.4)	**-0.09**<br>**(-0.61-0.49)***	0.56<br>(0.01 – 0.85)	0.08<br>(-0.50-0.60)	0.10<br>(-0.16-0.50)	**1.63**<br>**(1.16-2.77)***	1.94<br>(1.37 – 3.29)	7.37<br>(5.22-12.51)	1.91<br>(1.52 – 2.68)
	2	2.5<br>(0.3)	2.5<br>(0.9)	2.7<br>(0.3)	**-0.06**<br>**(-0.59-0.51)***	0.83<br>(0.52 – 0.95)	0.03<br>(-0.53-0.57)	0.08<br>(-0.17-0.48)	**1.36**<br>**(0.96-2.31)***	1.00<br>(0.71 – 1.69)	11.85<br>(8.40-20.13)	1.92<br>(1.53 – 2.70)
	3	2.6<br>(0.4)	2.5<br>(0.9)	2.8<br>(0.3)	**-0.02**<br>**(-0.57-0.54)***	0.82<br>(0.48-0.94)	**-0.09**<br>**(-0.61-0.49)***	0.05<br>(-0.19-0.45)	**1.37**<br>**(0.97 – 2.33)***	1.05<br>(0.74 – 1.78)	**1.39**<br>**(0.99-2.36)***	1.95<br>(1.55 – 2.74)

Kin	Interval	Mean (SD)			ICC(3,1) (95%CI)			ICC(3,1) mean (95%CI)	TE (stzd) (LL/UL)			TE (stzd) mean (LL/UL)
		**Sn. 1**	**Sn. 2**	**Sn. 3**	**2-1**	**3-1**	**3-2**		**2-1**	**3-1**	**3-2**	
	4	2.7 (0.4)	2.5 (0.9)	2.7 (0.4)	**-0.27 (-0.71-0.34)***	0.40 (-0.20-0.78)	0.05 (-0.51-0.59)	-0.04 (-0.25-0.34)	**1.57 (1.11-2.67)***	2.64 (1.87 – 4.49)	8.93 (6.33-15.16)	2.04 (1.62 – 2.85)
	5	2.7 (0.4)	2.5 (0.9)	2.7 (0.4)	0.01 (-0.54-0.56)	0.86 (0.58 – 0.96)	0.20 (-0.39-0.68)	0.23 (-0.07-0.61)	19.14 (13.36-32.50)	0.91 (0.65 – 1.55)	4.22 (2.99 – 7.17)	1.78 (1.42 – 2.50)
**RHy**	1	3.1 (0.4)	3.2 (0.2)	3.2 (0.2)	0.74 (0.32 – 0.92)	0.54 (-0.02-0.84)	0.48 (-0.10-0.81)	0.61 (0.30 – 0.85)	1.30 (0.92 – 2.20)	2.01 (1.43 – 3.42)	2.27 (1.61 – 3.86)	1.32 (1.05 – 1.84)
	2	3.0 (0.3)	3.2 (0.3)†	3.1 (0.2)	0.68 (0.20 – 0.90)	0.28 (-0.32-0.72)	0.44 (-0.14-0.80)	0.49 (0.16 – 0.79)	1.51 (1.07 – 2.56)	3.43 (2.43 – 5.83)	2.42 (1.71 – 4.10)	1.48 (1.18 – 2.07)
	3	3.0 (0.3)	3.2 (0.3)†	3.2 (0.1)b†	0.88 (0.64 – 0.96)	0.47 (-0.11-0.81)	0.51 (-0.06-0.83)	0.68 (0.39 – 0.88)	0.82 (0.58 – 1.40)	2.28 (1.62 – 3.87)	2.11 (1.50 – 3.59)	1.21 (0.96 – 1.69)
	4	3.1 (0.3)	3.2 (0.4)†	3.3 (0.2)	0.73 (0.30 – 0.91)	0.30 (-0.31-0.73)	0.47 (-0.12-0.81)	0.55 (0.23 – 0.82)	1.33 (0.94 – 2.26)	3.30 (2.34 – 5.60)	2.31 (1.64 – 3.93)	1.40 (1.11 – 1.96)
	5	3.2 (0.4)bc	3.2 (0.3)	3.3 (0.2)	0.84 (0.53 – 0.95)	0.41 (-0.18 –0.79)	0.40 (-0.20 –0.78)	0.61 (0.30 – 0.85)	0.98 (0.70 – 1.67)	2.57 (1.82 – 4.37)	2.66 (1.88 – 4.51)	1.31 (1.04 – 1.84)
**RHz**	1	2.2 (0.7)	2.0 (0.8)	2.2 (0.6)	0.66 (0.18 – 0.89)	0.61 (0.09 – 0.87)	0.29 (-0.31-0.73)	0.52 (0.20 – 0.81)	1.56 (1.10 – 2.65)	1.73 (1.23 – 2.94)	3.36 (2.38 – 5.70)	1.44 (1.14 – 2.01)
	2	2.2 (0.7)	1.9 (0.7)	2.2 (0.6)	0.76 (0.35 – 0.92)	0.61 (0.09 - 0.87)	0.31 (-0.29-0.74)	0.57 (0.25 – 0.83)	1.25 (0.89 – 2.13)	1.75 (1.24 – 2.97)	3.18 (2.25 – 5.40)	1.38 (1.10 – 1.93)
	3	2.3 (0.6)	2.1 (0.7)	2.2 (0.8)	0.66 (0.16 – 0.89)	0.71 (0.27 – 0.91)	0.25 (-0.36-0.70)	0.53 (0.20 – 0.81)	1.59 (1.13 – 2.70)	1.39 (0.99 – 2.37)	3.74 (2.65 – 6.35)	1.44 (1.14 – 2.01)
	4	2.4 (0.6)	2.2 (0.7)	2.3 (0.7)	0.77 (0.37 – 0.93)	0.70 (0.24 – 0.90)	0.30 (-0.30-0.73)	0.58 (0.26 – 0.83)	1.21 (0.86 – 2.06)	1.44 (1.02 – 2.45)	3.26 (2.31 – 5.53)	1.36 (1.08 – 1.90)
	5	2.5 (0.6)	2.1 (0.8)†	2.4 (0.7)	0.35 (-0.26-0.76)	0.73 (0.29 – 0.91)	0.29 (-0.31-0.73)	0.44 (0.11 – 0.76)	2.95 (2.09 – 5.00)	1.35 (0.96 – 2.29)	3.36 (2.38 – 5.71)	1.55 (1.23 – 2.17)

Kin = kinematics LHx = left hip flexion/extension, LHy = left hip abduction/adduction, LHz = left hip rotation, RHx = right hip flexion/extension, RHy = right hip abduction/adduction, RHz = right hip rotation, Sn = session; SD = standard deviation; ICC = intraclass correlation coefficient; CI = confidence interval; TE (stzd) = standardized typical error; LL = lower limit; UL = upper limit.

† < 0.05 vs. Sn. 1; ‡ < 0.05 vs. Sn. 2

a < 0.05 vs interval 1; b < 0.05 vs. interval 2; c < 0.05 vs. interval 3

Note: bolded* = raw data used to calculate typical error

Table S18. Reliability of the LyE for left and right knee kinematics

Kin	Interval	Mean (SD)			ICC$_{(3,1)}$ (95%CI)			ICC$_{(3,1)}$ mean (95%CI)	TE (stzd) (LL/UL)			TE (stzd) mean (LL/UL)
		**Sn. 1**	**Sn. 2**	**Sn. 3**	**2-1**	**3-1**	**3-2**		**2-1**	**3-1**	**3-2**	
**LKx**	1	1.9 (0.5)	2.0 (0.5)	1.8 (0.5)	0.53 (-0.03-0.84)	0.83 (0.52 – 0.95)	0.87 (0.61 – 0.96)	0.75 (0.50 – 0.91)	2.03 (1.44 – 3.45)	1.00 (0.71 – 1.70)	0.86 (0.61 – 1.47)	1.07 (0.85 – 1.50)
	2	1.8 (0.5)	1.9 (0.5)	1.7 (0.5)	0.56 (0.01 – 0.85)	0.82 (0.48 – 0.94)	0.88 (0.64 – 0.96)	0.76 (0.51 – 0.91)	1.94 (1.38 – 3.30)	1.05 (0.75 – 1.79)	0.82 (0.58 – 1.40)	1.06 (0.84 – 1.49)
	3	1.9 (0.6)	1.9 (0.5)	1.9 (0.5)	0.44 (-0.15-0.80)	0.82 (0.49 – 0.95)	0.79 (0.42 – 0.93)	0.69 (0.40 – 0.88)	2.46 (1.74 – 4.18)	1.04 (0.73 – 1.76)	1.15 (0.81 – 1.95)	1.19 (0.95 – 1.67)
	4	2.0 (0.5)	2.0 (0.5)	1.9 (0.5)	0.42 (-0.17-0.79)	0.86 (0.59 – 0.96)	0.80 (0.44 – 0.94)	0.71 (0.43 – 0.89)	2.51 (1.78 – 4.27)	0.90 (0.63 – 1.52)	1.11 (0.78 – 1.88)	1.16 (0.92 – 1.62)
	5	2.1 (0.5)$_{b}$	2.0 (0.6)$_{\dagger}$	1.8 (0.4)$_{\ddagger}$	0.47 (-0.11-0.81)	0.77 (0.38 – 0.93)	0.83 (0.51 – 0.95)	0.69 (0.40 – 0.88)	2.30 (1.63 – 3.91)	1.20 (0.85 – 2.04)	1.00 (0.71 – 1.70)	1.19 (0.95 – 1.67)
**LKy**	1	2.2 (0.5)	2.1 (0.5)	2.2 (0.6)	0.23 (-0.37-0.69)	0.73 (0.29-0.91)	0.14 (-0.45-0.64)	0.38 (0.05 – 0.72)	3.93 (2.79 – 6.68)	1.36 (0.96 – 2.30)	5.37 (3.80 – 9.12)	1.62 (1.29 – 2.27)
	2	2.1 (0.5)	2.0 (0.5)	2.1 (0.5)	0.21 (-0.38-0.69)	0.71 (0.27 – 0.91)	0.24 (-0.36-0.70)	0.40 (0.08 – 0.74)	4.08 (2.89 – 6.93)	1.39 (0.99 – 2.36)	3.76 (2.66 – 6.38)	1.59 (1.26 – 2.23)
	3	2.2 (0.5)	2.0 (0.6)	2.1 (0.5)	0.26 (-0.34-0.71)	0.64 (0.14 – 0.88)	0.26 (-0.34-0.71)	0.37 (0.05 – 0.72)	3.60 (2.55 – 6.11)	1.64 (1.16 – 2.78)	3.60 (2.55 – 6.11)	1.63 (1.30 – 2.28)
	4	2.3 (0.5)	2.0 (0.6)	2.1 (0.5)	0.27 (-0.33-0.71)	0.58 (0.04-0.86)	0.30 (-0.30-0.73)	0.37 (0.05 – 0.72)	3.53 (2.50 – 6.00)	1.87 (1.32 – 3.17)	3.24 (2.29 – 5.50)	1.63 (1.30 – 2.28)
	5	2.3 (0.4)	2.1 (0.6)	2.1 (0.6)	0.33 (-0.27-0.75)	0.58 (0.04 -0.86)	0.17 (-0.42-0.66)	0.35 (0.03 – 0.70)	3.03 (2.14 – 5.14)	1.84 (1.31 – 3.13)	4.63 (3.28 – 7.86)	1.65 (1.31 – 2.31)
**LKz**	1	1.4 (0.7)	1.5 (0.8)	1.4 (0.8)	0.29 (-0.32-0.72)	**-0.06 (-0.59-0.51)***	0.39 (-0.21-0.78)	0.22 (-0.08-0.61)	3.38 (2.39 – 5.73)	**1.50 (1.07-2.55)***	2.68 (1.90 – 4.55)	1.79 (1.42 – 2.51)
	2	1.3 (0.6)	1.4 (0.8)	1.3 (0.7)	0.27 (-0.34-0.71)	**-0.04 (-0.58-0.52)***	0.37 (-0.24-0.76)	0.21 (-0.09-0.60)	3.54 (2.51 – 6.01)	**1.36 (0.96-2.31)***	2.83 (2.01 – 4.81)	1.80 (1.43 – 2.52)
	3	0.9 (0.2)$_{ab}$	1.1 (0.4)$_{a}$	0.9 (0.3)$_{ab}$	0.53 (-0.04-0.84)	0.86 (0.58 – 0.96)	0.47 (-0.11-0.81)	0.57 (0.25 – 0.83)	2.06 (1.46 – 3.49)	0.90 (0.64 – 1.53)	2.30 (1.63 – 3.91)	1.38 (1.10 – 1.93)
	4	1.0 (0.2)$_{a}$	1.1 (0.4)	1.2 (0.6)	0.21 (-0.39-0.69)	0.38 (-0.22-0.77)	0.22 (-0.38-0.69)	0.27 (-0.04-0.65)	4.10 (2.90 – 6.96)	2.77 (1.96 – 4.71)	3.98 (2.82 – 6.75)	1.73 (1.38 – 2.43)
	5	0.9 (0.2)$_{ab}$	1.0 (0.2)$_{ab}$	1.3 (0.8)	0.77 (0.37 – 0.93)	0.29 (-0.31-0.73)	0.23 (-0.37-0.69)	0.29 (-0.03-0.66)	1.22 (0.86 – 2.06)	3.32 (2.35 – 5.63)	3.91 (2.77 – 6.64)	1.72 (1.37 – 2.41)
**RKx**	1	2.1 (0.5)	2.2 (0.3)	2.2 (0.5)	0.34 (-0.26-0.75)	0.66 (0.16 – 0.89)	0.26 (-0.35-0.71)	0.46 (0.13 – 0.77)	2.98 (2.11 – 5.06)	1.58 (1.12 – 2.69)	3.64 (2.58 – 6.19)	1.52 (1.21 – 2.13)
	2	2.1 (0.5)	2.1 (0.2)	2.2 (0.5)	0.27 (-0.33-0.72)	0.43 (-0.16-0.79)	0.21 (-0.39-0.68)	0.33 (0.01-0.69)	3.52 (2.49 – 5.97)	2.49 (1.77 – 4.23)	4.12 (2.92 – 7.00)	1.68 (1.33 – 2.35)
	3	2.1 (0.4)	2.1 (0.4)	2.2 (0.5)	0.37 (-0.23-0.77)	0.51 (-0.06-0.83)	0.15 (-0.44-0.65)	0.35 (0.03-0.70)	2.81 (1.99 – 4.77)	2.13 (1.51 – 3.61)	5.02 (3.55 – 8.52)	1.65 (1.32 – 2.32)

Kin	Interval	Mean (SD)			ICC$_{(3,1)}$ (95%CI)			ICC$_{(3,1)}$ mean (95%CI)	TE (stzd) (LL/UL)			TE (stzd) mean (LL/UL)
		Sn. 1	Sn. 2	Sn. 3	2-1	3-1	3-2		2-1	3-1	3-2	
	4	2.1<br>(0.4)	2.1<br>(0.3)	2.2<br>(0.5)	0.55<br>(-0.01-0.84)	0.41<br>(-0.19-0.78)	0.32<br>(-0.29-0.74)	0.41<br>(0.09 – 0.74)	1.98<br>(1.40 – 3.37)	2.61<br>(1.85 – 4.42)	3.14<br>(2.23 – 5.34)	1.58<br>(1.26 – 2.21)
	5	2.1<br>(0.4)	2.1<br>(0.3)	2.2<br>(0.6)	0.60<br>(0.07 – 0.87)	0.55<br>(0.00 – 0.85)	0.31<br>(-0.29-0.74)	0.47<br>(0.14 – 0.78)	1.79<br>(1.27 – 3.04)	1.96<br>(1.39 – 3.33)	3.19<br>(2.26 – 5.42)	1.51<br>(1.20 – 2.11)
RKy	1	2.4<br>(0.5)	2.6<br>(0.3)	2.6<br>(0.5)	0.29<br>(-0.32-0.72)	0.53<br>(-0.03-0.84)	0.49<br>(-0.09-0.82)	0.45<br>(0.12 – 0.77)	3.38<br>(2.40 – 5.74)	2.04<br>(1.45 – 3.47)	2.22<br>(1.57 – 3.77)	1.53<br>(1.22 – 2;14)
	2	2.4<br>(0.4)	2.5<br>(0.3)	2.5<br>(0.5)	0.13<br>(-0.45-0.64)	0.36<br>(-0.24-0.76)	0.42<br>(-0.17-0.79)	0.33<br>(0.01 – 0.69)	5.43<br>(3.84 – 9.21)	2.85<br>(2.02 – 4.85)	2.51<br>(1.78 – 4.27)	1.67<br>(1.33 – 2.35)
	3	2.4<br>(0.5)	2.5<br>(0.3)	2.6<br>(0.5)	0.79<br>(0.39 – 0.93)	0.82<br>(0.45 – 0.94)	0.67<br>(0.18 – 0.89)	0.77<br>(0.53 – 0.92)	1.13<br>(0.79 – 1.98)	1.04<br>(0.73 – 1.83)	1.55<br>(1.09 – 2.62)	1.03<br>(0.81 – 1.44)
	4	2.4<br>(0.5)	2.5<br>(0.3)	2.6<br>(0.5)	0.39<br>(-0.21-0.77)	0.66<br>(0.17 – 0.89)	0.53<br>(-0.03-0.84)	0.55<br>(0.23 – 0.82)	2.70<br>(1.92 – 4.59)	1.57<br>(1.11 – 2.66)	2.03<br>(1.44 – 3.45)	1.40<br>(1.11 – 1.96)
	5	2.4<br>(0.7)	2.5<br>(0.3)	2.6<br>(0.6)	0.33<br>(-0.27-0.75)	0.59<br>(0.05 – 0.86)	0.50<br>(-0.07-0.83)	0.49<br>(0.16 – 0.79)	3.05<br>(2.16 – 5.17)	1.83<br>(1.30 – 3.11)	2.15<br>(1.52 – 3.65)	1.48<br>(1.18 – 2.08)
RKz	1	1.3<br>(0.5)	1.2<br>(0.6)	1.1<br>(0.5)	0.36<br>(-0.25-0.76)	**-0.14**<br>**(-0.65-0.44)***	0.51<br>(-0.06-0.83)	0.27<br>(-0.04-0.64	2.89<br>(2.05 – 4.91)	**1.06**<br>**(0.75-1.80)***	2.13<br>(1.51 – 3.61)	1.74<br>(1.39 – 2.44)
	2	1.2<br>(0.5)	1.2<br>(0.6)	1.1<br>(0.4)	0.30<br>(-0.30-0.73)	**-0.15**<br>**(-0.65-0.44)***	0.47<br>(-0.11-0.81)	0.23<br>(-0.07-0.62)	3.27<br>(2.32 – 5.56)	**0.98**<br>**(0.69-1.66)***	2.28<br>(1.61 – 3.87)	1.78<br>(1.42 – 2.49)
	3	1.0<br>(0.1)	1.0<br>(0.1)	1.0<br>(0.1)	0.84<br>(0.53 – 0.95)	0.52<br>(-0.05-0.83)	0.60<br>(0.07 – 0.87)	0.68<br>(0.39 – 0.88)	0.98<br>(0.69 – 1.66)	2.09<br>(1.48 – 3.55)	1.77<br>(1.26 – 3.01)	1.20<br>(0.96 – 1.68)
	4	1.0<br>(0.2)[a]	1.0<br>(0.1)	1.0<br>(0.1)	0.84<br>(0.53 – 0.95)	0.75<br>(0.34 – 0.92)	0.87<br>(0.60 – 0.96)	0.81<br>(0.60 – 0.93)	0.98<br>(0.70 – 1.67)	1.28<br>(0.90 – 2.17)	0.88<br>(0.62 – 1.49)	0.95<br>(0.76 – 1.33)
	5	1.0<br>(0.1)	1.0<br>(0.2)	1.1<br>(0.4)	0.85<br>(0.57 – 0.96)	0.24<br>(-0.36-0.70)	0.16<br>(-0.43-0.65)	0.29<br>(-0.02-0.66)	0.92<br>(0.65 – 1.57)	3.77<br>(2.67 – 6.40)	4.95<br>(3.50 – 8.40)	1.72<br>(1.37 – 2.41)

Kin = kinematics LKx = left knee flexion/extension, LKy = left knee valgus/varum, LKz = left knee rotation, RKx = right knee flexion/extension, RKy = right knee valgus/varum, RKz = right knee rotation, Sn = session; SD = standard deviation; ICC = intraclass correlation coefficient; CI = confidence interval; TE (stzd) = standardized typical error; LL = lower limit; UL = upper limit.

† < 0.05 vs. Sn. 1; ‡ < 0.05 vs. Sn. 2.

a < 0.05 vs interval 1; b < 0.05 vs. interval 2

Note: bolded* = raw data used to calculate typical error

Table S19. Reliability of the LyE for left and right ankle kinematics

Kin	Interval	Mean (SD)			$ICC_{(3,1)}$ (95%CI)			$ICC_{(3,1)}$ mean (95%CI)	TE (stzd) (LL/UL)			TE (stzd) mean (LL/UL)
		**Sn. 1**	**Sn. 2**	**Sn. 3**	**2-1**	**3-1**	**3-2**		**2-1**	**3-1**	**3-2**	
**LAx**	1	1.0 (0.5)	0.9 (0.4)	0.9 (0.4)	0.82 (0.48 – 0.94)	0.70 (0.24 – 0.90)	0.78 (0.40 – 0.93)	0.77 (0.53 – 0.92)	1.05 (0.75 – 1.79)	1.44 (1.02 – 2.44)	1.18 (0.83 – 2.00)	1.04 (0.83 – 1.46)
	2	0.9 (0.5)	0.9 (0.4)	0.9 (0.3)	0.61 (0.08 – 0.87)	0.22 (-0.38-0.69)	0.34 (-0.26-0.75)	0.42 (0.09 – 0.74)	1.76 (1.25 – 2.98)	4.03 (2.86 – 6.85)	2.99 (2.12 – 5.08)	1.57 (1.25 – 2.21)
	3	0.9 (0.5)	1.0 (0.4)	0.9 (0.4)	0.74 (0.31 – 0.92)	0.19 (-0.40-0.67)	0.32 (-0.28-0.74)	0.45 (0.12 – 0.76)	1.32 (0.94 – 2.24)	4.33 (3.07 – 7.36)	3.13 (2.21 – 5.31)	1.53 (1.22 – 2.15)
	4	0.9 (0.5)	1.0 (0.4)	1.0 (0.3)	0.66 (0.17 – 0.89)	0.12 (-0.47-0.63)	0.33 (-0.27-0.75)	0.39 (0.06 – 0.72)	1.57 (1.11 – 2.67)	5.84 (4.14 – 9.92)	3.05 (2.16 – 5.18)	1.61 (1.28 – 2.26)
	5	0.9 (0.5)	1.0 (0.30	1.0 (0.4)	0.35 (-0.25-0.76)	0.42 (-0.18-0.79)	0.03 (-0.53-0.57)	0.22 (-0.07-0.61)	2.92 (2.07 – 4.97)	12.12 (8.58-20.57)	2.56 (1.81 – 4.35)	1.79 (1.42 – 2.50)
**LAy**	1	1.8 (0.5)	1.8 (0.4)	1.8 (0.5)	0.40 (-0.27-0.78)	0.63 (0.07 – 0.88)	0.32 (-0.28-0.74)	0.46 (0.12 – 0.77)	2.61 (1.83 – 4.59)	1.67 (1.16 – 2.93)	3.14 (2.22 – 5.33)	1.52 (1.19 – 2.12)
	2	1.7 (0.5)	1.8 (0.3)	1.7 (0.4)	0.44 (-0.15-0.80)	0.73 (0.29 – 0.91)	0.42 (-0.17-0.79)	0.55 (0.23 – 0.82)	2.45 (1.73 – 4.15)	1.35 (0.96 – 2.30)	2.54 (1.80 – 4.30)	1.40 (1.11 – 1.96)
	3	1.7 (0.6)	1.8 (0.4)	1.8 (0.5)	0.36 (-0.24-0.76)	0.66 (0.16 – 0.89)	0.52 (-0.04-0.83)	0.52 (0.20 – 0.80)	2.84 (2.01 – 4.82)	1.59 (1.12 – 2.69)	2.08 (1.47 – 3.53)	1.44 (1.15 – 2.02)
	4	1.8 (0.6)	1.8 (0.5)	1.9 (0.5)	0.66 (0.16 – 0.89)	0.74 (0.27 – 0.91)	0.74 (0.31 – 0.92)	0.71 (0.43 – 0.89)	1.58 (1.12 – 2.69)	1.31 (0.91 – 2.30)	1.31 (0.93 – 2.23)	1.15 (0.92 – 1.63)
	5	1.7 (0.5)	1.8 (0.5)	1.8 (0.5)	0.66 (0.17 – 0.89)	0.78 (0.40 – 0.93)	0.79 (0.42 – 0.93)	0.74 (0.49 – 0.91)	1.57 (1.12 – 2.67)	1.17 (0.83 – 1.99)	1.14 (0.81 – 1.94)	1.09 (0.87 – 1.52)
**LAz**	1	3.1 (0.3)	2.9 (0.3)†	2.9 (0.4)†	0.65 (0.11 – 0.88)	0.71 (0.22 – 0.91)	0.87 (0.62 – 0.96)	0.76 (0.50 – 0.91)	1.59 (1.11 – 2.78)	1.39 (0.97 – 2.45)	0.86 (0.61 – 1.46)	1.06 (0.83 – 1.48)
	2	3.0 (0.2)	2.9 (0.3)	2.8 (0.3)	0.60 (0.08 – 0.87)	0.70 (0.24 – 0.90)	0.89 (0.67 – 0.97)	0.75 (0.50 – 0.91)	1.77 (1.25 – 3.01)	1.44 (1.02 – 2.44)	0.78 (0.55 – 1.33)	1.07 (0.85 – 1.50)
	3	3.0 (0.2)	2.9 (0.3)	2.9 (0.3)	0.67 (0.19 – 0.89)	0.72 (0.28 – 0.91)	0.75 (0.34 – 0.92)	0.72 (0.45 – 0.90)	1.54 (1.09 – 2.61)	1.36 (0.96 – 2.31)	1.26 (0.89 – 2.14)	1.13 (0.90 – 2.14)
	4	3.0 (0.3)	3.0 (0.3)	2.9 (0.3)	0.58 (0.04 – 0.86)	0.82 (0.48 – 0.94)	0.62 (0.10 – 0.87)	0.67 (0.38 – 0.88)	1.86 (1.32 – 3.16)	1.05 (0.74 – 1.78)	1.72 (1.22 – 2.91)	1.22 (0.97 – 1.71)
	5	3.1 (0.2)	3.0 (0.3)	3.0 (0.4)	0.38 (-0.22-0.77)	0.67 (0.18 – 0.89)	0.69 (0.21 – 0.90)	0.60 (0.29 – 0.85)	2.77 (1.96 – 4.70)	1.56 (1.10 – 2.64)	1.49 (1.05 – 2.52)	1.32 (1.05 – 1.85)
**RAx**	1	1.0 (0.5)	1.0 (0.4)	1.0 (0.5)	0.38 (-0.22-0.77)	0.46 (-0.13-0.81)	0.56 (0.01 – 0.85)	0.47 (0.14 – 0.77)	2.74 (1.94 – 4.65)	2.36 (1.67 – 4.00)	1.92 (1.36 – 3.27)	1.51 (1.20 – 2.12)
	2	1.0 (0.4)	1.0 (0.4)	1.0 (0.4)	0.73 (0.29 – 0.91)	0.50 (-0.08-0.82)	0.53 (-0.04-0.84)	0.59 (0.27 – 0.84)	1.35 (0.96 – 2.29)	2.18 (1.54 – 3.70)	2.06 (1.46 – 3.50)	1.35 (1.07 – 1.89)
	3	1.0 (0.3)	1.0 (0.5)	1.0 (0.3)	0.49 (-0.15-0.82)	0.26 (-0.42-0.71)	0.50 (-0.07-0.83)	0.44 (0.11 – 0.76)	2.19 (1.53 – 3.84)	3.57 (2.49 – 6.26)	2.17 (1.53 – 3.68)	1.54 (1.21 – 2.15)

	4	1.0 (0.4)	1.0 (0.5)	0.9 (0.4)	0.46 (-0.12-0.81)	0.46 (-0.13-0.81)	0.51 (-0.05-0.83)	0.48 (0.15 – 0.78)	2.33 (1.65 – 3.96)	2.35 (1.66 – 3.98)	2.11 (1.49 – 3.58)	1.49 (1.19 – 2.09)
	5	1.0 (0.3)	1.0 (0.5)	1.0 (0.4)	0.67 (0.18 – 0.89)	0.89 (0.65 – 0.97)	0.79 (0.43 – 0.94)	0.78 (0.54 – 0.92)	1.55 (1.10 – 2.64)	0.80 (0.57 – 1.37)	1.13 (0.80 – 1.92	1.02 (0.81 – 1.43)
**RAy**	1	2.1 (0.4)	2.2 (0.4)	2.1 (0.4)	0.80 (0.44 – 0.94)	0.24 (-0.36-0.70)	0.19 (-0.40-0.67)	0.40 (0.07 – 0.73)	1.11 (0.79 – 1.89)	3.79 (2.68 – 6.43)	4.38 (3.10 – 7.44)	1.60 (1.27 – 2.24)
	2	2.1 (0.4)	2.1 (0.3)	2.1 (0.4)	0.77 (0.37 – 0.93)	**-0.02 (-0.57-0.54)***	**-0.01 (-0.56-0.54)***	0.20 (-0.09-0.59)	1.22 (0.86 – 2.07)	**0.82 (0.58-1.40)***	**0.78 (0.55-1.32)***	1.81 (1.44 – 2.53)
	3	2.2 (0.3)	2.2 (0.3)	2.2 (0.5)	0.70 (0.23 – 0.90)	0.54 (-0.02-0.84)	0.64 (0.14 – 0.88)	0.62 (0.31 – 0.85)	1.45 (1.03 – 2.46)	2.02 (1.43 – 3.42)	1.63 (1.15 – 2.76)	1.30 (1.04 – 1.83)
	4	2.2 (0.4)	2.1 (0.3)	2.1 (0.4)	0.39 (-0.21-0.77)	0.50 (-0.07-0.83)	0.27 (-0.33-0.72)	0.40 (0.07-0.74)	2.70 (1.92 – 4.59)	2.16 (1.53 – 3.68)	3.49 (2.47 – 5.93)	1.59 (1.27 – 2.23)
	5	2.2 (0.4)	2.2 (0.4)	2.1 (0.5)	0.55 (-0.01-0.84)	0.28 (-0.32-0.72)	0.52 (-0.05-0.83)	0.44 (0.11-0.76)	1.98 (1.40 – 3.36)	3.41 (2.42 – 5.80)	2.10 (1.49 – 3.56)	1.55 (1.23 – 2.17)
**RAz**	1	3.1 (0.4)	3.1 (0.4)	3.1 (0.4)	0.48 (-0.10-0.82)	0.62 (0.09 – 0.87)	0.71 (0.26 – 0.91)	0.61 (0.30 – 0.85)	2.25 (1.60 – 3.83)	1.73 (1.22 – 2.93)	1.41 (1.00 – 2.40)	1.32 (1.05 – 1.84)
	2	3.0 (0.3)	2.9 (0.3)	3.0 (0.4)	0.57 (0.03 – 0.86)	0.63 (0.12 – 0.88)	0.71 (0.25 – 0.91)	0.64 (0.34 – 0.86)	1.88 (1.33 – 3.19)	1.67 (1.19 – 2.84)	1.42 (1.01 – 2.42)	1.27 (1.01 – 1.77)
	3	3.1 (0.3)	3.0 (0.3)	3.0 (0.4)	0.46 (-0.13-0.81)	0.48 (-0.10-0.82)	0.76 (0.35 – 0.92)	0.58 (0.26 – 0.83)	2.36 (1.68 – 4.02)	2.24 (1.59 – 3.81)	1.25 (0.89 – 2.13)	1.36 (1.09 – 1.31)
	4	3.1 (0.3)	3.1 (0.3)	3.1 (0.3)	0.84 (0.54-0.95)	**-0.10 (-0.62-0.48)**	0.01 (-0.55-0.56)	0.30 (-0.02-0.67)	0.97 (0.69-1.65)	**0.68 (0.48-1.15)***	21.29 (15.08-36.14)	1.71 (1.36-2.39)
	5	3.1 (0.3)	3.1 (0.3)	3.1 (0.4)	0.44 (-0.14-0.80)	0.50 (-0.07-0.83)	0.64 (0.14 – 0.88)	0.54 (0.22 – 0.81)	2.42 (1.71 – 4.10)	2.16 (1.53 – 3.67)	1.63 (1.15 – 2.77)	1.41 (1.13 – 1.98)

Kin = kinematics LAx = left ankle dorsi/plantarflexion, LAy = left foot progression, LAz = left foot rotation, RAx = right ankle dorsi/plantaflexion, RAy = right foot progression, RAz = right foot rotation, Sn = session; SD = standard deviation; ICC = intraclass correlation coefficient; CI = confidence interval; TE (stzd) = standardized typical error; LL = lower limit; UL = upper limit.

† < 0.05 vs. Sn. 2; ‡ < 0.05 vs. Sn. 3.

Note: bolded* = raw data used to calculate typical error
